# Cell Wall Hydrolases in Bacteria: Insight on the Diversity of Cell Wall Amidases, Glycosidases and Peptidases Toward Peptidoglycan

**DOI:** 10.3389/fmicb.2019.00331

**Published:** 2019-02-28

**Authors:** Aurore Vermassen, Sabine Leroy, Régine Talon, Christian Provot, Magdalena Popowska, Mickaël Desvaux

**Affiliations:** ^1^Université Clermont Auvergne, INRA, MEDiS, Clermont-Ferrand, France; ^2^BioFilm Control SAS, Saint-Beauzire, France; ^3^Department of Applied Microbiology, Faculty of Biology, Institute of Microbiology, University of Warsaw, Warsaw, Poland

**Keywords:** bacterial cell wall, peptidoglycan (PG) hydrolases, protein modules, cell wall binding domains, bacterial division and growth, cell lysis, cell wall remodeling

## Abstract

The cell wall (CW) of bacteria is an intricate arrangement of macromolecules, at least constituted of peptidoglycan (PG) but also of (lipo)teichoic acids, various polysaccharides, polyglutamate and/or proteins. During bacterial growth and division, there is a constant balance between CW degradation and biosynthesis. The CW is remodeled by bacterial hydrolases, whose activities are carefully regulated to maintain cell integrity or lead to bacterial death. Each cell wall hydrolase (CWH) has a specific role regarding the PG: (i) cell wall amidase (CWA) cleaves the amide bond between N-acetylmuramic acid and L-alanine residue at the N-terminal of the stem peptide, (ii) cell wall glycosidase (CWG) catalyses the hydrolysis of the glycosidic linkages, whereas (iii) cell wall peptidase (CWP) cleaves amide bonds between amino acids within the PG chain. After an exhaustive overview of all known conserved catalytic domains responsible for CWA, CWG, and CWP activities, this review stresses that the CWHs frequently display a modular architecture combining multiple and/or different catalytic domains, including some lytic transglycosylases as well as CW binding domains. From there, direct physiological and collateral roles of CWHs in bacterial cells are further discussed.

## Introduction

The first bacterial cell wall hydrolase (CWH) was discovered in 1921 by the Scottish bacteriologist Sir Alexander Fleming, who is well known for his 1928 discovery of the antibiotic penicillin (Fleming, 1929). He had observed “a remarkable bacteriolytic element,” which caused bacterial lysis on an agar plate and which he called “lysozyme” in 1922. Lysozyme cleaves the β-(1-4)-glycosidic bond between N-acetylmuramic acid (MurNAc) and N-acetylglucosamine (GlcNAc) in peptidoglycan (PG) (Chipman et al., 1967). This hydrolytic enzyme has served as a model in protein biochemistry and its contribution to antibacterial defense is well recognized.

**Figure 1:**
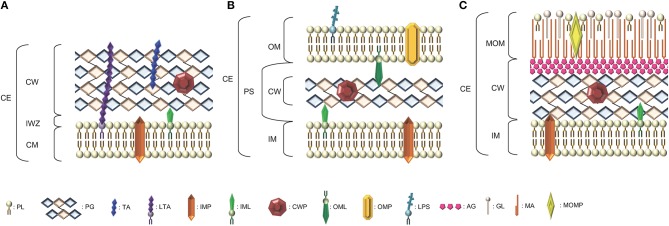
Schematic representation of the diversity of arrangements for bacterial cell envelopes. **(A)** Parietal monoderm (CW-monoderm) contains one cell membrane with a thick peptidoglycan layer and teichoic and lipoteichoic acids. **(B)** Lipopolysaccharidic diderm (LPS-diderm) bacteria are enveloped by two membranes with a thin peptidoglycan layer between the inner and outer membranes. **(C)** Mycolate diderm (myco-diderm) bacteria have a cytoplasmic membrane and a peculiar outer membrane constituted of mycolic acid, also called mycomembrane, with a cell wall in-between constituted of a peptidoglycan fraction and an arabinogalactan fraction. CE, cell envelope; CW, cell wall; CM, cytoplasmic membrane; OM, outer membrane; PS, periplasmic space; IM, inner membrane; IWZ, inner wall zone; MOM, mycolic outer membrane; PL, Phospholipid; PG, peptidoglycan; TA, teichoic acid; LTA, lipoteichoic acid; IMP, integral/inner membrane protein; IML, inner membrane lipoprotein; CWP, cell wall protein; OML, outer membrane lipoprotein; OMP, outer membrane protein; LPS, lipopolysaccharide; AG, arabinogalactan; GL, glycolipid; MA, mycolic acid; MOMP, mycolic outer membrane protein.

The bacterial cell wall (CW) is a complex arrangement of macromolecules that varies depending on the species of bacteria and whether they are parietal monoderm bacteria (archetypal Gram-positive bacteria), lipopolysaccharidic diderm bacteria (archetypal Gram-negative bacteria) or mycolate diderm bacteria (archetypal acid-fast bacteria) (Desvaux et al., 2018; Figure 1). While the PG is the major polymer of the CW, teichoic and lipoteichoic acids as well as other macromolecular components like polysaccharides, polyglutamate or proteins can also be (Neuhaus and Baddiley, 2003; Dramsi et al., 2008; Vollmer et al., 2008a). In resisting to internal turgor pressure to maintain bacterial cell integrity and shape, the CW is essential to bacterial growth under various environmental conditions (Weidel and Pelzer, 1964; Vollmer et al., 2008a). Besides, it needs to be remodeled to accommodate cell elongation and division for proper bacterial growth. In CW-monoderm bacteria, the CW is an interface between the cell and its environment and participates in interactions with abiotic surfaces, bacteriophages and eukaryotic host cells (Silhavy et al., 2010).

Depending on the CW bonds they specifically cleave, CWHs can be discriminated into three types of enzymes, namely amidases, glycosidases or peptidases (Rigden et al., 2003; Fenton et al., 2010; Szweda et al., 2012). CWHs were historically described as lysins and classified as endolysins, exolysins and/or autolysins based on their origin and role (Vollmer et al., 2008b; Schmelcher et al., 2012). In fact, endolysins referred to bacteriophage encoded CWHs degrading the CW of the host bacterium upon activation of the lytic cycle. Exolysins referred to secreted bacterial CWHs aiming at killing bacterial cells of different species or even strains within the same species. Rather than bacterial cell lysis, autolysins referred to hydrolases mainly involved in CW remodeling in the course of bacterial cell division (Vollmer et al., 2008b; Vollmer, 2012). However, advances in biochemical and structural characterization indicate that these lysins include amidases, glycosidases and peptidases. In function, endolysins are similar to exolysins and autolysins, except that these two latter hydrolases are not encoded by bacteriophages (Schmelcher et al., 2012). Moreover, a so-called autolysin can induce cell lysis (and not just CW remodeling) when its expression is not tightly controlled (Typas et al., 2012). For these reasons, in this manuscript, we will favor the term CWHs over lysins, and describe them with respect to their catalytic activity into CW amidases, CW glycosidases and/or CW peptidases rather than endolysins, exolysins and autolysins. This nomenclature highlights the importance of the modular architecture of the polypeptide for its function. Since the PG is the only CW component targeted by CWHs characterized to date, they can be interchangeably called PG hydrolases (PGHs).

After a brief reminding of the CW structure and organization in bacteria, the different conserved domains related to bacterial CWHs are here exhaustively overviewed in order to further stress the diversity of their modular architecture and their physiological relevance is further discussed.

## The Cell Wall: Composition, Structure and Organization

Bacteria have developed a complex cell envelope, which helps them to survive in various environments (Vollmer et al., 2008a; Silhavy et al., 2010). The CW is a macromolecule with an elastic-plastic behavior that defines the shape of the bacterium (Amir et al., 2014). It enables the bacterial cell to resist lysis as a result of its high intracellular osmotic pressure. In CW-monoderm bacteria, the cytoplasmic membrane is surrounded by a thick CW (10–20 layers thick) composed of PG, to which secondary polymers are covalently attached (e.g., teichoic acids (Figure 1; Vollmer et al., 2008a); in addition, lipoteichoic acids are anchored to the cytoplasmic membrane via their diacylglycerol moiety. Some polysaccharides, namely teichuronic acids or polyglutamate, and surface proteins can be covalently or non-covalently linked to the PG. Actually, the totality of the molecular components found at the bacterial cell surface refers to the surfaceome and its protein subset constitutes the proteosurfaceome (Desvaux et al., 2018). When displayed at the surface of CW-monoderm bacteria, proteins can either be associated with the cytoplasmic membrane, i.e., IMPs (integral membrane proteins) or lipoproteins, or with the CW (Popowska and Markiewicz, 2004; Desvaux et al., 2006). At the CW, proteins can either be covalently anchored (LPXTG-proteins) or specifically associated with some CW components through weak interactions thanks to the presence of conserved motifs, e.g., LysM (lysin motif), CWB1 (CW binding repeat of type 1) or PGB1 (peptidoglycan binding domain of type 1) (Desvaux et al., 2006, 2018). In the cell envelope of LPS-diderm bacteria, the CW is located between the cytoplasmic membrane (then also called inner membrane) and the outer membrane, i.e., within the periplasmic space. It consists of a thin layer of PG (only 1–3 layers thick) linked to some lipoproteins anchored to the outer membrane (Figure 1; Torti and Park, 1976).

**Figure 2:**
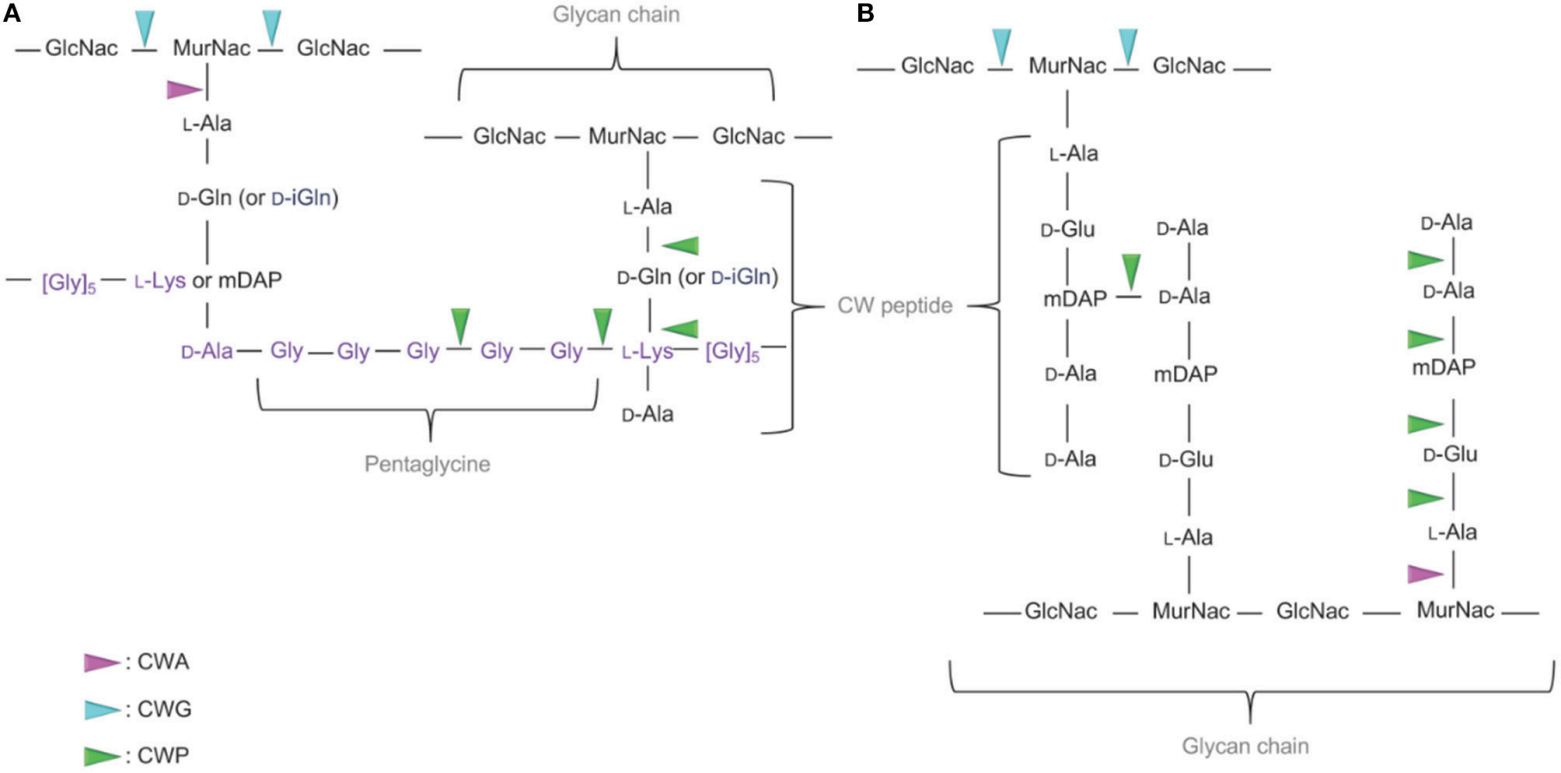
Targets of the CWHs in CW-monoderm and LPS-diderm bacteria. **(A)** In the CW of CW-monoderm bacteria, the alternating subunits of N-acetylglucosamine (GlcNac) and N-acetylmuramic acid (MurNac) are amide linked to the alanine of the wall peptide alanine, glutamine or isoglutamine, meso-diaminopimelic acid (mDAP) or lysine and alanine. In *S. aureus*, the pentaglycine cross bridge is linked to the alanine of the CW peptide. **(B)** In the CW of LPS-diderm bacteria, the alternating subunits of N-acetylglucosamine (GlcNac) and N-acetylmuramic acid (MurNac) are amide linked to the alanine of tetrapeptide alanine, glutamine, mDAP and alanine. N-acetylglucosaminidase hydrolyses the glycan component of the cell wall on the reducing side of the GlcNAc. In contrast, the lysozyme hydrolyses the glycan component of the cell wall on the reducing side of the MurNAc. Likewise, N-acetylmuramidases cleave the same bond but form N-acetyl-1,6-anhydro-muramyl intermediates during cleavage. N-acetylmuramoyl-L-alanine amidase cleaves a critical amide bond between the glycan moiety (MurNAc) and the peptide moiety (L-alanine) of the peptidoglycan. Peptidase cleaves an amide bond between two amino acids (depicted in green). CWA, CW amidase; CWG, CW glycosidase; CWP, CW peptidase.

The molecular organization, variations in the primary structure and description of the different types of the PG in bacteria has been extensively described in dedicated reviews readily available for in-depth details (Schleifer and Kandler, 1972; Turner et al., 2014; Desvaux et al., 2018). In CW-monoderm bacteria, the PG polymer results from the cross-linking of glycan strands through peptide stems. While peptide stems originate from the lactyl moiety of the MurNAc saccharide, the glycan strands are composed of alternating MurNAc and GlcNAc saccharides (Figure 2; Bourhis and Werts, 2007; Johnson et al., 2013). In the stem peptide, the first two residues of are generally L-Ala and D-Gln or iGln, whereas D-Ala is typically the last one (Humann and Lenz, 2009). In many rod-shaped CW-monoderm bacteria, e.g., *Listeria* or *Bacillus*, a meso-diaminopimelate (mDAP) constitutes the third residue of the stem peptide. In cocci species, though, a lysine residue is found at this position (Vollmer et al., 2008a). The *Staphylococcus aureus* PG comprises 20 or more layers of linear glycan chains with alternating MurNAc and GlcNAc, and a L-Ala-D-iGln-L-Lys-D-Ala stem peptide (Dmitriev et al., 2004). To connect PG chains, a pentaglycine interpeptide branches off the amino group of the L-Lys of the stem peptide to the D-Ala in the position of a neighboring chain (Figure 2). In *S. aureus*, the PG chains have a maximum length of 23–26 disaccharide units but the majority of chains ranges between 3 and 10 (Boneca et al., 2000). Actually, most of the diversity in the PG composition occurs with the nature of the crosslinking of the stem peptides (Schleifer and Kandler, 1972).

In LPS-diderm bacteria, the composition of PG is quite similar with alternating MurNAc and GlcNAc (Figure 2). However, a 1,6-anhydro modification is present at the end of the strand in MurNAc residue (Vollmer and Holtje, 2004). Of note, such modification can also be detected in some CW-monoderm bacteria, like *B. subtilis* (Atrih et al., 1999). The L-Ala-D-Glu-mDAP-D-Ala-D-Ala stem peptides is linked to the lactyl group of MurNAc (Figure 2). Most cross-links result from the mDAP at position 3 of one stem peptide with the D-Ala at position 4 of a second stem peptide of a neighboring glycan strand (Vollmer and Holtje, 2004). In *Escherichia coli*, the length of glycan chains is around 25–35 disaccharide units in average but the length distribution is quite broad (Harz et al., 1990).

In myco-diderm bacteria, the mycolic outer membrane (MOM) or mycomembrane has been intensively investigated (Niederweis et al., 2010; Jackson, 2014) and the CW located between the MOM and the IM is constituted of two main fractions, the PG and arabinogalactan (AG), which are covalently attached (Alderwick et al., 2015). The AG is actually connected to mycolic acids at the MOM then forming the so-called mycolyl-arabinogalactan-peptidoglycan complex. Regarding the PG and compared to CW-monoderm or LPS-diderm bacteria, much less information is available but it is generally assumed its synthesis is similar to that of *E. coli* (Schleifer and Kandler, 1972; Van Heijenoort, 2001; Alderwick et al., 2015). Briefly, the PG in myco-diderm bacteria is composed of alternating GlcNAc and MurNAc, linked in a β-1,4 configuration (Alderwick et al., 2015). Besides MurNAc, N-glycolyl derivatives of the muramic acid (MurNGly) are also present as a result of the oxidation of the N-acetyl group to a N-glycolyl group. Regarding the stem peptides, the proportion of cross-linking is also significantly higher in myco-diderm bacteria compared to LPS-diderm bacteria as observed for *Mycobacterium* species vs. *E. coli* (Alderwick et al., 2015).

In both CW-monoderm and LPS-diderm bacteria, modifications to the basic PG structure such as N-glycosylation, O-acetylation and/or N-deacetylation occur frequently and many of them are species-specific (Markiewicz and Popowska, 2011). Moreover, in response to environmental conditions, the PG structure of a given bacterium may also change. Such modifications could enhance resistance to antibiotics and host degradative enzymes targeting the CW. Modifications to the basic PG structure occur at several levels, namely in the disaccharide backbone, the bridge regions, and the peptide stem (Humann and Lenz, 2009).

## Cell Wall Hydrolases in Bacteria

The classification of a CWH as CW amidase, CW glycosidase and/or CW peptidase is associated with the presence of conserved catalytic domains respective to these different enzymatic activities (Alcorlo et al., 2017). These functionally important domains can be identified in proteins following searches against Interpro (IPR) (Mitchell et al., 2019), the most renown and reliable integrative protein signature databank regrouping different specialist member databases, such as Pfam (Finn et al., 2016), SMART (Letunic and Bork, 2018) or CDD (Marchler-Bauer et al., 2017). In fact, the identification of a conserved motif based on a probabilistic match against HMM (hidden Markov model) or even PSSM (position-specific scoring matrix) profiles is more effective, relevant and robust than a percentage of identity or similarity against regular expressions (Nagl, 2003). The cleavage sites of the PG by the different CWHs are shown in Figure 2. To date, only CWHs involved in the degradation of PG have been reported, and consequently they can be synonymously and more precisely called PGHs, at least until CWHs targeting other components of the CW are reported and characterized. Regarding the structure of solved CWHs readers can refer to recent review for further in-depth knowledge (Alcorlo et al., 2017; Broendum et al., 2018).

### Cell Wall Amidases (CWA, E.C.3.5.1)

**Figure 3:**
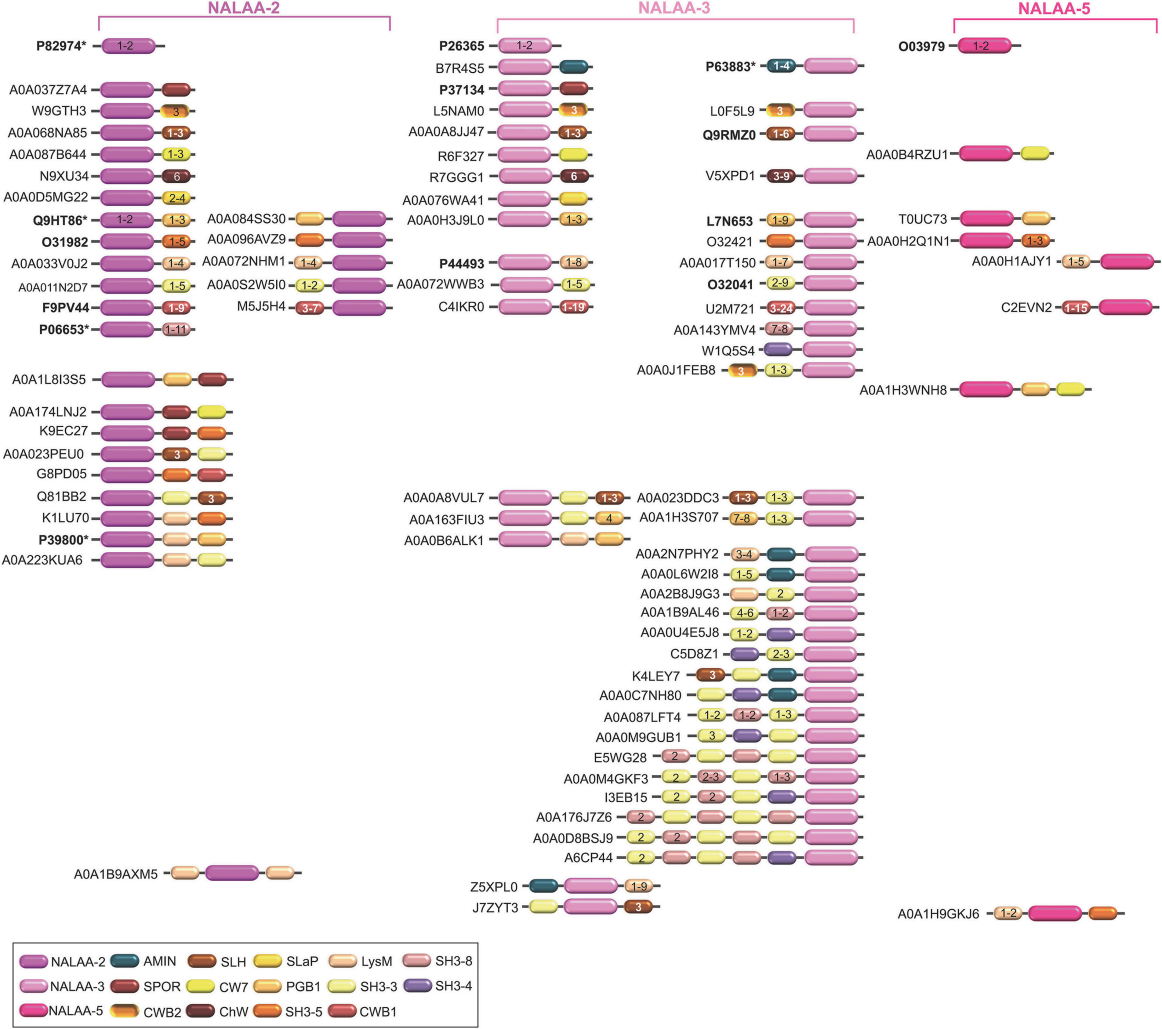
Schematic representation of the diversity of modular architectures of CWHs acting as CWA only. For each modular organization, a UniProt identifier is provided as a representative. Bold identifier indicates that at least one representative protein has been functionally characterized (including the given identifier). Asterisk indicates that at least one representative has been structurally characterized (including the given identifier).

CWAs actually correspond to N-acetylmuramoyl-L-alanine amidases (NALAAs) and can also be called PG amidases (PGAs) or amidases in the scientific literature (Young, 1992; Shockman et al., 1996). They hydrolyse the amide bond separating the glycan strand from the stem peptide, that is between the MurNAc and L-alanine residues (Holtje, 1995; Vollmer et al., 2008b). In bacteria, three different types of catalytic domains are currently reported as responsible for a NALAA activity, namely (i) N-acetylmuramoyl-L-alanine amidase of type 2 (NALAA-2; IPR002502), (ii) N-acetylmuramoyl-L-alanine amidase of type 3 (NALAA-3; IPR002508), and (iii) N-acetylmuramoyl-L-alanine amidase of type 5 (NALAA-5; IPR008044) (Table 1 and Figure 3).

**Table 1:** Conserved catalytic domains responsible for cell wall amidase (CWA) activity in bacterial cell wall hydrolases (CWHs).

**Catalytic domain^a^**	**Abbreviation**	**InterPro**	**Other databases^b^**	**Structure^c^**	**Additional catalytic domain^a, d^**	**Cell wall binding domain^e^**	**Bacteria^f^**
Cell wall amidase	CWA						
N-acetylmuramoyl-L-alanine amidase of type 2	NALAA-2	IPR002502	PF01510 SM00644 SSF55846 G3DSA:3.40.80.10 CD06583	1J3G, 2Y28, 2Y2B, 2Y2C, 2Y2D, 2Y2E, 4IVV, 4X36, 3D2Z, 5CTV, 4BOL, 4BJ4, 4BPA, 4BXJ, 4BXD, 3RDR, 3HMB, 4BXE, 2BH7, 2WKX, 2D2Y, 3D2Z, 4OLS	GHF-73 GHF-25	PGB1, SPOR, SH3-3, SH3-5, SH3-8, SLAP, LysM, CW7, SLH, CWB1, CWB2, ChW	Proteobacteria (*Pseudomonas, Acinetobacter*), Actinobacteria (*Streptomyces, Mycobacterium, Micromonspora*), Firmicutes (*Clostridium, Bacillus, Lactobacillus, Paenibacillus, Staphylococcus, Enterococcus, Selenomonas*), Bacteroidetes (*Flavobacterium, Chryseobacterium, Bacteroides, Prevotella, Chitinophaga*), Cyanobacteria (*Synechococcus*), Chloroflexi (Chloroflexus), Fusobacteria (*Fusobacterium, Leptotrichia*)
					CHAP NlpC/P60 PM15 PM23 PM74		
					SLT1 TG		
N-acetylmuramoyl-L-alanine amidase of type 3	NALAA-3	IPR002508	PF01520 SM00646 G3DSA:3.40.630.40 CD02696	4BIN, 4KNK, 4KNL, 3LAT, 4EPC	GHF-73	LysM, AMIN, PGB1, SPOR, SH3-3, SH3-4, SH3-8, SLAP, CWB2, SLH, CWB1, CW7, ChW	Firmicutes (*Clostridium, Bacillus, Paenibacillus, Lactobacillus, Eubacterium, Desulfatomaculum*), Proteobacteria (*Pseudomonas, Vibrio, Sphingomonas*), Bacteroidetes (*Flavobacterium, Bacteroides, Prevotella*), Actinobacteria (*Mycobacterium, Streptomyces, Corynebacterium*), Cyanobacteria (*Nostoc, Calothrix, Synechococcus*)
					CHAP NlpC/P60 PM15 PM23 PM74		
					SLT1		
N-acetylmuramoyl-L-alanine amidase of type 5	NALAA-5	IPR008044	PF05382	n.d.	GHF-73 GHF-24	SH3-5, PGB1, LysM, CW7, CWB1	Firmicutes (*Lactobacillus, Streptococcus, Enterococcus, Clostridium, Anaerococcus, Fructobacillus, Lactococcus*), Proteobacteria (*Acinetobacter*), Actinobacteria (*Bifidobacterium*)
					CHAP PM23		

a *Coloured highlighting indicate the type of cell wall hydrolase (CWH), namely cell wall amidase (CWA; pink), cell wall glycosidase (CWG; cyan) or cell wall peptidase (CWP; green)*.

b *Databases used to construct the InterPro entry, namely Pfam (PF), SMART (SM), Conserved Domain database (CD), Prosite (PS), Prints (PR), SuperFamily (SF), Protein Information Resource System (PIRSF), TIGRFam (TIGR), HAMAP (MF), CATH-Gene3D (G3DSA)*.

c *Identifier from the Protein DataBase (PDB). n.d.: structure not determined*.

d *Additional catalytic domains that can be associated with the catalytic domain under consideration in a given monopolypeptide along the modular architecture of the protein. Catalytic domains corresponding to CWA are shaded in pink, to CWG in blue, and to CWP in green. Unshaded catalytic domains correspond to non-hydrolytic enzymes, namely N-acetylmuramidases (lytic transglycosylases), namely the TG (transglycosylase; IPR010618), RlpA (rare lipoprotein A; IPR034718), MltG (membrane-bound lytic transglycosylase of type G; IPR003770), SleB (Spore cortex-lytic enzyme of Bacillus; IPR011105), SLT1 (soluble lytic murein transglycosylase of type 1; IPR008258) or SLT2 (IPR031304) domains*.

e *Additional cell wall binding domains that can found along the modular architecture of a monopolypeptide. LysM: lysin motif (IPR018392), PGB1: peptidoglycan binding domain of type 1 (IPR002477), PGB2 (IPR014927), PGB3 (IPR018537), PGB4 (IPR022029), SLH: S-layer homology domain (IPR001119), LysM: lysin motif (IPR018392), SPOR: sporulation-related (IPR007730), CWB1: cell wall binding repeat of type 1 (IPR018337), CWB2 (IPR007253), ChW: clostridial hydrophobic repeat with a conserved W residue (IPR006637), CW7: cell wall binding domain of Cpl-7 (IPR013168), SLaP: S-layer protein (IPR024968), AMIN: N-terminal nonamidase (IPR021731), SH3-1: sarcoma [src] homology 3 of type 1 (PF00018), SH3-2 (PF07653), SH3-3 (PF08239), SH3-4 (PF06347), SH3-5 (PF08460), SH3-6 (PF12913), SH3-7 (PF12914), SH3-8 (PF13457) and SH3-9 (PF14604)*.

f *Main phyla of the kingdom Bacteria and examples of some of the main bacterial genera (in brackets and in italics) with bacterial genomes encoding protein harboring the CWA catalytic domain as recorded in InterPro and Pfam databases*.

#### N-acetylmuramoyl-L-alanine amidase of Type 2 (NALAA-2)

The NALAA-2 domain is about 125 amino acids long (IPR002502). *E. coli* AmpD is one of the most investigated CWHs with a NALAA-2 domain (Holtje et al., 1994; Van Heijenoort, 2001). This enzyme rapidly cleaves 1,6-anhydro-MurNAc-L-Ala bonds in MurNAc-tri and tetrapeptides. AmiD (amidase D) has a broader substrate specificity in cleaving both 1,6-anhydro-MurNAc-L-Ala and MurNAc-L-Ala bonds (Pennartz et al., 2009). In some *Bacillus* species, a truncated version of this domain can be found, that is the NALAA-2C (N-acetylmuramoyl-L-alanine amidase domain of type 2 C-terminal; IPR021976), which remains poorly understood (Yang et al., 2013). The structure of NALAA-2 catalytic domain has been extensively investigated (Table 1; Firczuk and Bochtler, 2007; Alcorlo et al., 2017). The catalysis is zinc-dependent and occurs in a L-shape cavity where the Zn^2+^ cation is located at the intersection of the region for the glycan binding and the region for the peptide stem binding (Lee et al., 2013; Martinez-Caballero et al., 2013). NALAA-2 has an open and closed conformation as the enzyme activation involves important structural rearrangements at the substrate and peptide-stem binding sites (Liepinsh et al., 2003; Carrasco-Lopez et al., 2011).

#### N-acetylmuramoyl-L-alanine amidase of Type 3 (NALAA-3)

The NALAA-3 domain is approximately 175 amino acids long (IPR002508). In *E. coli*, the NALAA-3 AmiA and AmiC were shown to result in the release of the L-Ala-D-Glu-mDAP tripeptide and L-Ala-D-Glu-mDAP-D-Ala tetrapeptide. Like NALAA-2, NALAA-3 is a zinc-dependent amidase, which structure has been solved (Zoll et al., 2012; Rocaboy et al., 2013; Buttner et al., 2014).

#### N-acetylmuramoyl-L-alanine amidase of Type 5 (NALAA-5)

The NALAA-5 domain is around 140 amino acids long (IPR008044). Dp-1, a bacteriophage of *Streptococcus pneumoniae* belonging to Siphoviridae family, encodes a NALAA-5 CW degrading enzyme (Lopez et al., 1981; Garcia et al., 1983). It was the first pneumococcal CWH to be purified and biochemically characterized as a CWA, which was further demonstrated to require choline for full enzymatic activity (Garcia et al., 1983). No structural information is as yet available for this CWA family.

### Cell Wall Glycosidases (CWG, EC 3.2.1)

**Figure 4:**
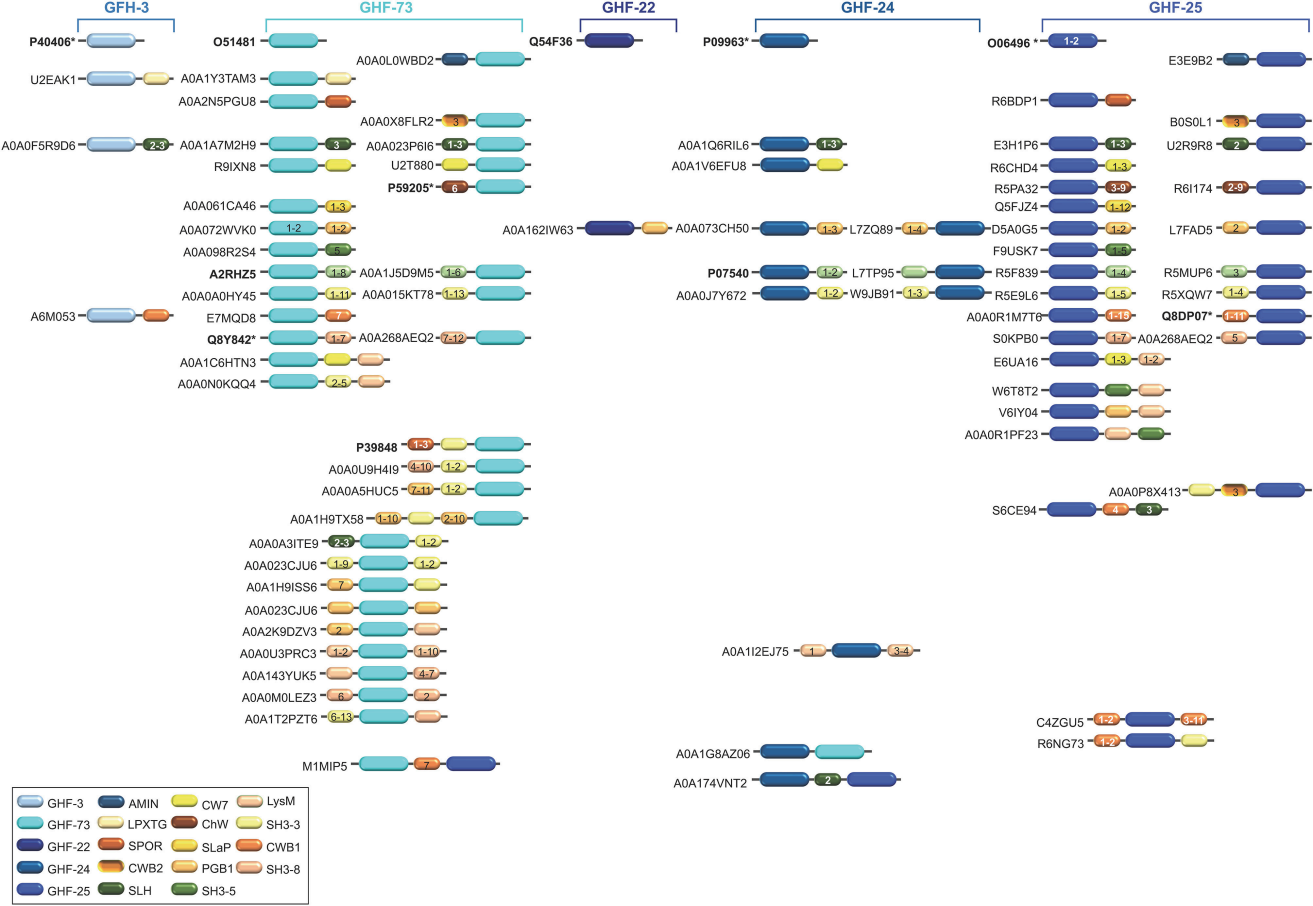
Schematic representation of the diversity of modular architectures of CWHs acting as CWG only. For each modular organization, a UniProt identifier is provided as a representative. Bold identifier indicates that at least one representative protein has been functionally characterized (including the given identifier). Asterisk indicates that at least one representative has been structurally characterized (including the given identifier).

Glycosidases catalyse the hydrolysis of the glycosidic linkage (O-, N-, and S-linked), leading to the formation of a glucide hemiacetal or hemiketal (Holtje, 1995; Vollmer et al., 2008b). Glycosidases can also be called glycoside or glycosyl hydrolases. Over the years, the number of families of glycosidases has grown steadily and currently there are 135 families according to the CAZyme (Carbohydrate Active Enzymes) database (CAZy; http://www.cazy.org). CW glycosidases (CWGs), also called PG glycosidases (PGGs), can be broadly differentiated into (i) N-acetylglucosaminidases, and (ii) lysozymes (Table 2 and Figure 4).

**Table 2:** Conserved catalytic domains responsible for cell wall glycosidase (CWG) activity in bacterial cell wall hydrolases (CWHs).

**Catalytic domain^a^**	**Abbreviation**	**InterPro**	**Other databases^b^**	**Structure^c^**	**Additional catalytic domain^a, d^**	**Cell wall binding domain^e^**	**Bacteria^f^**
Cell wall glycosidase	CWG						
**N-acetylglucosaminidase**							
Glycoside hydrolase family 3	GHF-3	IPR002772 IPR001764	PF01915 SSF52279 G3DSA:3.40.50.1700 PF00933 PR00133 G3DSA:3.20.20.300	3NVD, 3BMX, 4GYJ, 4GYK, 2OXN, 1TR9, 3LK6, 3TEV, 4GVG, 4GVI, 4GVF, 4GVH, 3NVD	PM15	SLH, CWBD1, LPXTG	Actinobacteria (*Streptomyces, Bifidobacterium, Microbacterium, Cellulomonas*), Bacteroidetes (*Flavobacterium, Prevotella, Bacteroides, Chryseobacterium, Mucilaginibacter*), Firmicutes (*Clostridium, Paenibacillus, Bacillus, Enterococcus, Butyrivibrio, Lactobacillus*), Proteobacteria (*Sphingomonas, Pseudomonas, Sphingobium*)
Glycoside hydrolase family 73	GHF-73	IPR002901	PF01832 SM00047	2Q2W, 4KNK, 4KNL, 3LAT, 4EPC, 3FI7, 5JQC, 2ZYC, 3VWO, 3K3T	NALAA-2 NALAA-3 NALAA-5	LysM, AMIN, SLH, SH3-3, SH3-5, SH3-8, CWB1, CWBD2, PGB1, ChW, LPXTG	Firmicutes (*Lactobacillus, Enterococcus, Clostridium, Bacillus, Staphylococcus, Paenibacillus, Streptococcus, Carnobacterium*), Proteobacteria (*Pseudomonas, Vibrio, Pseudoalteromonas, Shewanella, Burkholderia, Massilia*), Bacteroidetes (*Flavobacterium, Chryseobacterium, Capnocytophaga, Parabacteroides, Pedobacter, Sphingobacterium, Mucilaginibacter, Chitinophaga, Lewinella*), Actinobacteria (*Actinomyces, Olsenella, Tessaracoccus*), Cyanobacteria (*Nostoc, Calothrix*),
					GHF-24 GHF-25		
					CHAP NlpC/P60 PM23		
					MltG		
**Lysozymes**							
Glycoside hydrolase family 22	GHF-22	IPR001916	PF00062	n.d.	none	PGB1	Proteobacteria (*Labilithrix*), Actinobacteria (*Frankia*), Firmicutes (*Enterococcus*)
Glycoside hydrolase family 24	GHF-24	IPR002196	PF00959	2ANV, 2ANX	NALAA-5	PGB1, SH3-3, CW7, LysM, SLH	Proteobacteria (*Burkholderia, Parabulkholderia, Pantoea, Erwinia, Xenorhabdus, Acinetobacter, Sodalis, Pseudomonas, Vibrio, Pseudoalteromonas, Bartonella, Rhizobium, Sphingomonas, Novosphingobium, Erythrobacter, Azospirillum, Paracoccus*), Cyanobacteria (*Nostoc, Calthrix, Leptolyngbyaceae, Microcoleaceae, Oscillatoriaceae*), Bacteroidetes (*Chryseobacterium, Prevotella, Sphingobacterium, Chitinophaga*), Firmicutes (*Clostridium, Paenibacillus*), Plantomycetes (*Fimbriiglobus, Phycisphaerae*), Actinobacteria (*Mycobacterium, Corynebacterium*), Acidobacteria (*Granulicella, Bryocella, Candidatus*), Fusobacteria (*Fusobacterium, Bryocella*)
					GHF-73 GHF-25		
					CHAP PM23 PM15		
Glycoside hydrolase family 25	GHF-25	IPR002053	PF01183	2WWD, 2WW5, 2WWD, 5JIP	NALAA-2	LysM, AMIN, SLH, PGB1, SH3-3, SH3-5, SH3-8, CWB1, CWB2, CW7, SPOR, SLAP, ChW	Firmicutes (*Lactobacillus, Clostridium, Streptococcus, Enterococcus, Ruminococcus, Butyrivibrio, Eubacterium, Bacillus, Erysipelatoclostridium*), Actinobacteria (*Streptomyces, Corynebacterium, Rhodococcus, Nocardia, Arthrobacter, Micromonospora, Amycolatopsis, Bifidobacterium, Saccharopolyspora*), Proteobacteria (*Mesorhizobium, Rhizobium, Sphingomonas, Acinetobacter*), Bacteroidetes (*Flavobacterium, Chryseobacterium, Prevotella, Bacteroides, Pedobacter, Chitnophaga*), Cyanobacteria (*Planktothrix, Nostoc*), Chloroflexi (*Chloroflexi, Anaerolineaceae, Pelolinea*)
					GHF-73 GHF-24		
					CHAP PM15 PM23		

a *Colored highlighting indicate the type of cell wall hydrolase (CWH), namely cell wall amidase (CWA; pink), cell wall glycosidase (CWG; cyan) or cell wall peptidase (CWP; green)*.

b *Databases used to construct the InterPro entry, namely Pfam (PF), SMART (SM), Conserved Domain database (CD), Prosite (PS), Prints (PR), SuperFamily (SF), Protein Information Resource System (PIRSF), TIGRFam (TIGR), HAMAP (MF), CATH-Gene3D (G3DSA)*.

c *Identifier from the Protein DataBase (PDB). n.d.: structure not determined*.

d *Additional catalytic domains that can be associated with the catalytic domain under consideration in a given monopolypeptide along the modular architecture of the protein. Catalytic domains corresponding to CWA are shaded in pink, to CWG in blue, and to CWP in green. Unshaded catalytic domains correspond to non-hydrolytic enzymes, namely N-acetylmuramidases (lytic transglycosylases), namely the TG (transglycosylase; IPR010618), RlpA (rare lipoprotein A; IPR034718), MltG (membrane-bound lytic transglycosylase of type G; IPR003770), SleB (Spore cortex-lytic enzyme of Bacillus; IPR011105), SLT1 (soluble lytic murein transglycosylase of type 1; IPR008258) or SLT2 (IPR031304) domains*.

e *Additional cell wall binding domains that can found along the modular architecture of a monopolypeptide. LysM: lysin motif (IPR018392), PGB1: peptidoglycan binding domain of type 1 (IPR002477), PGB2 (IPR014927), PGB3 (IPR018537), PGB4 (IPR022029), SLH: S-layer homology domain (IPR001119), LysM: lysin motif (IPR018392), SPOR: sporulation-related (IPR007730), CWB1: cell wall binding repeat of type 1 (IPR018337), CWB2 (IPR007253), ChW: clostridial hydrophobic repeat with a conserved W residue (IPR006637), CW7: cell wall binding domain of Cpl-7 (IPR013168), SLaP: S-layer protein (IPR024968), AMIN: N-terminal nonamidase (IPR021731), SH3-1: sarcoma [src] homology 3 of type 1 (PF00018), SH3-2 (PF07653), SH3-3 (PF08239), SH3-4 (PF06347), SH3-5 (PF08460), SH3-6 (PF12913), SH3-7 (PF12914), SH3-8 (PF13457) and SH3-9 (PF14604)*.

f *Main phyla of the kingdom Bacteria and examples of some of the main bacterial genera (in brackets and in italics) with bacterial genomes encoding protein harboring the CWA catalytic domain as recorded in InterPro and Pfam databases*.

#### N-acetylglucosaminidases

N-acetylglucosaminidases hydrolyse the glycosidic bond in different oligosaccharide substrates including the CW, chitin and N-glycans by cleaving specifically between N-acetyl-β-D-glucosamine residues and contiguous monosaccharides (Karamanos, 1997). To date, two domains have been reported as involved in N-acetylglucosaminidase activity, the (i) glycosyl hydrolase family 3 (GHF-3; IPR001764), and (ii) glycosyl hydrolase family 73 (GHF-73; IPR001764) (Table 2).

##### Glycosyl hydrolase family 3 (GHF-3)

The GHF-3 domain is about 300 amino acids long (IPR001764) with a conserved Asp-His dyad is involved in CW lysis (Litzinger et al., 2010). NagZ (N-acetylglucosaminidase Z) from *E. coli* is one of the best characterized GHF-3 CWGs, which cleaves the GlcNAc-(1-4)-1,6-anhydro-MurNAc disaccharide (Cheng et al., 2000; Votsch and Templin, 2000; Van Heijenoort, 2001). It is active on both monomer and dimer muropeptides and is specific for the β linkage. In *B. subtilis*, the NagZ ortholog requires CWA AmiE to release MurNAc efficiently by sequential hydrolysis of muropeptides (Litzinger et al., 2010). NagZ from *Vibrio cholerae* was the first GHF-3 enzyme to be solved at structural level (Stubbs et al., 2007). As in many glycosyl hydrolases, it features a (β/α)_8_ barrel fold, known as a TIM barrel, with a large cavity containing the active catalytic site formed of an Asp-Glu dyad. However, structural investigation with NagZ from *B. subtilis* revealed a unique Asp-His catalytic mechanism (Litzinger et al., 2010). Interestingly, this enzyme contains an additional GHF-3 C-terminal domain (IPR002772) but the catalysis appears to rely only on N-terminal TIM-barrel domain (Varghese et al., 1999). Besides, the loop, where the catalytic His is located, undergoes significant structural changes during the binding of the substrate and catalysis (Bacik et al., 2012).

##### Glycosyl hydrolase family 73 (GHF-73)

The GHF-73 domain is around 80 amino acids long (IPR002901) where the active catalytic site is formed by a tetrad of Tyr-Ala-Thr-Asp amino acid residues together with a highly conserved glutamic acid residue (Huard et al., 2004; Inagaki et al., 2009). The first structure of a GHF-73 enzyme was obtained from Auto (autolysin) from *Listeria monocytogenes* (Bublitz et al., 2009). It revealed the mechanism of autoinhibition via the occlusion of the substrate binding cleft by an N-terminal α-helix. As confirmed in the other solved structures for this glycosyl hydrolase family (Hashimoto et al., 2009; Maruyama et al., 2010), Glu appears as the catalytic residue in the active site of LytB in *S. pneumoniae* (Bai et al., 2014).

#### Lysozymes

Lysozymes (also sometimes referred as N-acetylmuramidases) and lytic transglycosylases (LTGs) cleave the same β1,4-glycosidic bond but in two different ways. While its hydrolysis by lysozymes result in a product with a terminal reducing MurNAc residue without a ring, LTGs are not hydrolases but cleave the bond between MurNAc and GlcNAc with the concomitant formation of a 1,6-anhydro ring at the MurNAc residue (Herlihey and Clarke, 2017). Lysozymes are generally categorized into at least five different classes, i.e., C (chicken type), G (goose type), P (Bacteriophage lambda), F (fungi), and B (bacteria) (Jolles, 1996; Pei and Grishin, 2005; Vollmer et al., 2008b; Callewaert and Michiels, 2010; Callewaert et al., 2012). In bacteria, lysozymes remain poorly characterized but three conserved domains are currently recognized, i.e., the (i) glycosyl hydrolase family 22 (GHF-22; IPR001916), (ii) glycosyl hydrolase family 24 (GHF-24; IPR002190) and (iii) glycosyl hydrolase family 25 (GHF-25; IPR002053) (Table 2).

##### Glycoside hydrolase family 22 (GHF-22)

The GHF-22 domain is about 120 amino acids long (IPR001916) and corresponds to lysozymes of type C almost uniquely found in species of the superkingdom Eukaryota (Mckenzie, 1996; Callewaert and Michiels, 2010). However, homologous sequences were recently identified in some bacterial species (Yamamoto et al., 2014; D'angelo et al., 2016) (Table 2).

##### Glycoside hydrolase family 24 (GHF-24)

The GHF-24 domain is approximately 110 amino acids long (IPR002190) and corresponds to phage lysozymes (type P), which structure has been solved only for the bacteriophage P22 lysozyme (Mooers and Matthews, 2006). Together with GHF-22, the function and activity of GHF-24 enzymes still await to be investigated in bacteria.

##### Glycoside hydrolase family 25 (GHF-25)

The GHF-25 domain is about 180 amino acid long (IPR002053) and corresponds to the lysozymes of type B such as Cpl-1, produced by the phage Cp-1, which specifically lyses several serotypes of *S. pneumoniae* (Perez-Dorado et al., 2007; Doehn et al., 2013). LytC from *S. pneumoniae* was the first GHF-25 enzyme whose structure has been solved (Perez-Dorado et al., 2010). Conformational changes appear to play a key role in PG hydrolysis, especially in the control of the enzymatic activity. In SleM from *Clostridium perfringens*, dimerisation was demonstrated to be of great importance for the enzymatic activity, most certainly by facilitating the positioning of substrate respective to the catalytic site (Al-Riyami et al., 2016).

### Cell Wall Peptidases (CWPs, EC 3.4)

**Figure 5:**
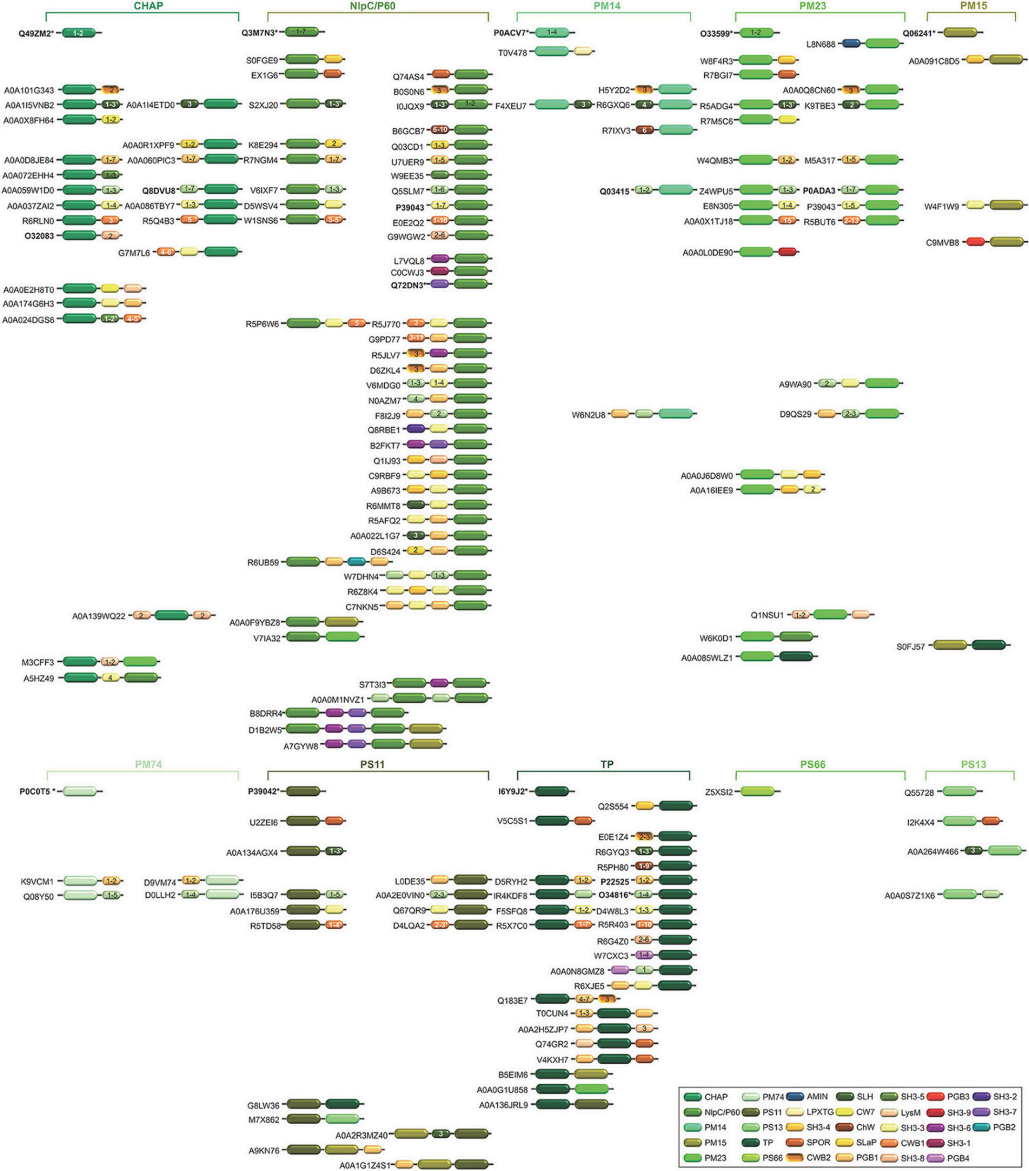
Schematic representation of the diversity of modular architectures of CWHs acting as CWP only. For each modular organization, a UniProt identifier is provided as a representative. Bold identifier indicates that at least one representative protein has been functionally characterized (including the given identifier). Asterisk indicates that at least one representative has been structurally characterized (including the given identifier).

A CW peptidase (CWP) cleaves the amide bonds between amino acids in PG (Holtje, 1995). CWPs, also called PG peptidases (PGPs), can be discriminated between endopeptidases or carboxypeptidases depending on their substrate specificity, as they respectively cleave within the PG peptide or remove the C-terminal amino acids. While D,L- and L,D-peptidases cleave between L- and D-amino acids, D,D-peptidases cleave between two D-amino acids (Smith et al., 2000). To date, 10 different types of domains have been reported for CWPs, the (i) cysteine histidine-dependent amidohydrolases/peptidases (CHAP; IPR007921), (ii) new lipoprotein C/protein of 60-kDa (NlpC/P60; IPR000064), (iii) peptidase M14 (PM14; IPR005073), (iv) peptidase M15 (PM15; IPR000755), (v) peptidase M23 (PM23; IPR016047), (vi) peptidase M74 (PM74; IPR005073), (vii) peptidase S11 (PS11; IPR001967), (viii) peptidase S13 (PS13; IPR000667), (ix) transpeptidase (TP, IPR005490), and (x) peptidase S66 (PS66; IPR003507) (Table 3 and Figure 5).

**Table 3:** Conserved catalytic domains responsible for cell wall peptidase (CWP) activity in bacterial cell wall hydrolases (CWHs).

**Catalytic domain^a^**	**Abbreviation**	**InterPro**	**Other databases^b^**	**Structure^c^**	**Additional catalytic domain^a, d^**	**Cell wall binding domain^e^**	**Bacteria^f^**
Cell wall peptidase	CWP						
Cysteine histidine-dependent amidohydrolases/peptidase	CHAP	IPR007921	PF05257 PS50911	2K3A, 4CSH, 4CT3, 4OLK	NALAA-2 NALAA-3 NALAA-5	LysM, PGB1, SH3-3, SH3-5, SH3-8, SLH, CW7, SLAP, CWB1, CWB2	Firmicutes (*Clostridium, Staphylococcus, Streptococcus, Enterococcus, Lactococcus, Lactobacillus, Paenibacillus, Ruminococcus*), Actinobacteria (*Bifidobacterium, Streptomyces, Nonomuraea, Arthrobacter, Mycobacterium, Rhodococcus, Actinomadura, Nocardioides*), Proteobacteria (*Sphingomonas, Sphingobium, Sphingopyxis, Nonosphingobium, Bradyrhizobium, Rhizobium, Acetobacter, Brevindimonas, Burkholderia, Klebsiella, Psychrobacter, Moraxella*), Bacteroidetes (*Sphingobacterium, Flavobacterium, Mucilaginibacter*), Chloroflexi (*Ktedonobacter*), Cyanobacteria (*Scytonema*)
					GHF-73 GHF-24		
					NlpC/P60 PM23		
					SleB SLT1 SLT2		
New lipoprotein C/protein of 60-kDa	NlpC/P60	IPR000064	PF00877 G3DSA:3.90.1720.10	2HBW, 3NPF, 3H41, 3M1U, 4FDY, 4HPE	NALAA-2 NALAA-3,	PGB1, PGB2, LysM, SH3-1, SH3-2, SH3-3, SH3-4, SH3-5, SH3-6, SH3-7, SH3-8, SLH, SPOR, SLAP, CWB2, CWB1, ChW	Actinobacteria (*Streptomyces, Mycobacterium, Corynebacterium, Rhodococcus, Nocardia, Gordonia, Arthrobacter, Micromonospora, Amycolatopsis, Actinomyces, Geodermatophilus, Blastococcus, Pseudonocardia*), Firmicutes (*Bacillus, Lactobacillus, Paenibacillus, Clostridium, Lachnoclostridium, Eubacterium, Ruminiclostridium, Faecalibacterium, Lysinibacullus*), Proteobacteria (*Pseudomonas, Helicobacter, Desulfovibrio, Rhizobium, Bodetella, Paraburkholderia, Burkholderia, Acidovorax, Variovorax, Vibrio, Halomonas, Xenorhabdus, Pantoea*), Bacteroidetes (*Flavobacterium, Chryseobacterium, Bacteroides, Pedobacter, Sphinngobacterium, Mucilagenobacterium*), Cyanobacteria (*Synechococcus, Nostoc*), Spirochaetes (*Treponema, Leptospira*), Chloroflexi (*Chloroflexus, Chloroflexi*),
					GHF-73		
					CHAP PM15 PM23		
					SleB SLT1 SLT2 TG		
Peptidase M14	PM14	IPR000834	PF00246 PS00132 PR00765 SM00631	5HXD	none	LysM, PGB1, LPXTG, SLH, CWB2, ChW	Proteobacteria (*Sphingomonas, Pseudoalteromonas, Shewanella, Vibrio, Pseudomonas, Lysobacter, Brevindimonas*), Bacteroidetes (*Flavobacterium, Chryseobacterium, Polaribacter, Cellulophaga, Pedobacter, Algoriphagus, Chitinophaga, Spirosoma, Maribacter*), Actinobacteria (*Streptomyces, Micromonospora, Monomuraea, Nocardioides, Lentzea, Amycolatopsis*), Firmicutes (*Bacillus, Paenibacillus, Clostridium, Ruminococcus, Halobacillus, Virgibacillus*), Ignavibacteriae (*Ignavibacterium*), Chloroflexi (*Anaerolineaceae, Anaerolineae*)
Peptidase M15	PM15	IPR000755	PF01427 MF01924 PIRSF026671	1R44, 4OXD, 4OX5, 4MUR, 4MUS, 4MUT, 4OAK	NALAA-2 NALAA-3	PGB3, SH3-3, SH3-4	Proteobacteria (*Legionella, Pseudomonas, Halomonas, Burkholderia, Bradyrhizobium, Desulfovibrio, Calobacter*), Actinobacteria (*Streptomyces, Mycobacterium, Micronospora, Amycolatopsis, Nonomuraea, Nocardioides*), Bacteroidetes (*Flavobacterium, Prevotella, Bacteroides, Pedobacter, Chitinophaga, Hymenobacter*), Cyanobacteria (*Synechococcus, Calothrix, Nostoc, Planktothrix*)
					GHF-3 GHF-25		
					NlpC/P60 PS11 TP		
					SleB		
Peptidase M23	PM23	IPR016047	PF01551	1QWY, 4ZYB	NALAA-2 NALAA-3 NALAA-5 GHF-24	LysM, AMIN, SH3-3, SH3-4, SH3-9, CWB1, CWB2, SLH, SPOR, CW7, PGB1	Proteobacteria (*Pseudomonas, Helicobacter, Desulfovibrio, Sphingomonas, Novosphingobium, Rhizobium, Vibrio, Pseudoalteromonas, Burkholderia*), Actinobacteria (*Streptomyces, Mycobacterium, Arthrobacter, Micronospora, Corynebacterium*), Firmicutes (*Bacillus, Clostridium, Paenibacillus, Eubacterium, Ruminococcus*), Bacteroidetes (*Flavobacterium, Bacteroides, Prevotella, Chryseobacterium*), Cyanobacteria (*Synechococcus, Calothrix, Nostoc*), Spirochaetes (*Leptospira, Treponema*), Chloroflexi (*Chloroflexus, Anaerolinea*), Deinococcus-Thermus (*Meiothermus, Deinococcus*)
					GHF-73		
					NlpC/P60 TP		
					SleB SLT1 SLT2 TG RlpA		
Peptidase M74	PM74	IPR005073	PF03411 MF01623, PIRSF018455	1U10, 1TPZP	NALAA-2 NALAA-3	LysM, PGB1	Proteobacteria (*Bradyrhizobium, Ochrobactrum, Brucella, Methylobacterium, Mesorhizobium, Rhizobium, Paracoccus, Rhodobacter, Roseovarius, Sulfitobacter, Escherichia, Klebsiella, Salmonella, Pantoea, Serratia, Sorangium, Bdellovibrio*), Actinobacteria (*Streptomyces*), Bacteroidetes (*Fluviicola*)
					SLT1		
Peptidase S11	PS11	IPR001967	PF00768	1SKF, 1ES4, 1ES5, 1ES2, 3A3J, 1TVF, 3IT9	PM15 TP PS13	SPOR, CWB1, LysM, SLH, SH3-3	Proteobacteria (*Pseudomonas, Acinetobacter, Vibrio, Rhizobium, Mesorhizobium, Sphingomonas, Bordetella, Burkholderia, Marinobacter, Bradyrhizobium, Devosia, Acetobacter*), Firmicutes (*Clostridium, Bacillus, Eubacterium, Lachnoclostridium, Ruminococcus, Paenibacillus, Lactobacillus, Streptococcus, Ruminiclostridium*), Actinobacteria (*Mycobacterium, Mycobacterium, Microbacterium, Micromonospora, Amycolatopsis, Streptomyces, Olsenella*), Chloroflexi (*Ktedonobacter*), Fusobacteria (*Fusobacterium, Leptotrichia*), Verrucomicrobia (*Akkermansia, Roseibacillus*)
					SLT1		
Peptidase S13	PS13	IPR000667	PF02113 TIGR00666 PR00922	3A3D, 2EX2,	PS11	SPOR, LysM, SLH	Proteobacteria (*Pseudomonas, Vibrio, Halomonas, Marinobacter, Legionella, Sphingomonas, Paraburkholderia, Caballeronia, Acidovorax, Bordetella*), Actinobacteria (*Streptomyces, Mycobacterium, Arthrobacter, Corynebacterium, Micromonospora, Nocardioides, Bifidobacterium, Actinomyces, Rhodococcus), Bacteroidetes (Prevotella, Chryseobacterium, Pedobacter, Bacteroides, Sphingobacterium, Mucilaginibacter, Chitinophaga*), Cyanobacteria (*Synechococcus, Cyanothece, Calothrix, Nostoc, Leptolyngbya*), Firmicutes (*Bacillus, Virgibacillus, Lysinibacillus*), Acidobacteria (*Chloracidobacterium, Granulicella*), Ignavibacteriae (*Melioribacter*), Planctomycetes (*Singulisphaera, Gemmata*)
Peptidase S66	PS66	IPR003507	PF02016 PIRSF028757	n.d.	none	none	Proteobacteria (Burkholderia, Paraburkholderia, Limnohabitans, Achromobacter, Acidovorax, Pseudoalteromonas, Escherichia, Klebsiella, Salmonella, Desulfobulbus), Firmicutes (Clostridium, Eubacterium, Veillonella, Megasphaera, Megamonas), Actinobacteria (*Streptomyces, Nonomuraea, Microbacterium, Micromonospora*)
Transpeptidase	TP	IPR005490	PF03734	4XZZ, 4Y4V, 3VYP, 5D7H, 5DU7, 4GSQ, 5DVP, 5DUJ	PM15 PM23 PS11	PGB1, PGB4, LysM, SLH, SH3-3, SH3-4, SH3-8, SPOR, CWB1, ChW, CWB2	Proteobacteria (*Pseudomonas, Rhizobium, Mesorhizobium, Bradyrhizobium, Methylobacterium, Sphinogomonas, Loktanella, Paracoccus, Roseovarius, Geobacter*), Actinobacteria (*Streptomyces, Mycobacterium, Micronospora, Corynebacterium, Rhodococcus, Pseudonorcardia*), Firmicutes (*Clostridium, Lactobacillus, Bacillus, Paenibacillus, Lachnoclostridium, Propionispora*), Bacteroidetes (*Bacteroides, Flavobacterium, Pedobacter, Hymenobacter, Chitinophaga), Prevotella, Parabacteroides, Porphyromonas, Odoribacter, Dysgonomonas*), Cyanobacteria (*Synechococcus, Nostoc*), Verrucomicrobia (*Akkermansia, Roseibacillus*), Chloroflexi (*Anaerolinea, Chloroflexus, Ktedonobacter*)
					SLT1 TG		

a *Colored highlighting indicate the type of cell wall hydrolase (CWH), namely cell wall amidase (CWA; pink), cell wall glycosidase (CWG; cyan) or cell wall peptidase (CWP; green)*.

b *Databases used to construct the InterPro entry, namely Pfam (PF), SMART (SM), Conserved Domain database (CD), Prosite (PS), Prints (PR), SuperFamily (SF), Protein Information Resource System (PIRSF), TIGRFam (TIGR), HAMAP (MF), CATH-Gene3D (G3DSA)*.

c *Identifier from the Protein DataBase (PDB). n.d.: structure not determined*.

d *Additional catalytic domains that can be associated with the catalytic domain under consideration in a given monopolypeptide along the modular architecture of the protein. Catalytic domains corresponding to CWA are shaded in pink, to CWG in blue, and to CWP in green. Unshaded catalytic domains correspond to non-hydrolytic enzymes, namely N-acetylmuramidases (lytic transglycosylases), namely the TG (transglycosylase; IPR010618), RlpA (rare lipoprotein A; IPR034718), MltG (membrane-bound lytic transglycosylase of type G; IPR003770), SleB (Spore cortex-lytic enzyme of Bacillus; IPR011105), SLT1 (soluble lytic murein transglycosylase of type 1; IPR008258) or SLT2 (IPR031304) domains*.

e *Additional cell wall binding domains that can found along the modular architecture of a monopolypeptide. LysM: lysin motif (IPR018392), PGB1: peptidoglycan binding domain of type 1 (IPR002477), PGB2 (IPR014927), PGB3 (IPR018537), PGB4 (IPR022029), SLH: S-layer homology domain (IPR001119), LysM: lysin motif (IPR018392), SPOR: sporulation-related (IPR007730), CWB1: cell wall binding repeat of type 1 (IPR018337), CWB2 (IPR007253), ChW: clostridial hydrophobic repeat with a conserved W residue (IPR006637), CW7: cell wall binding domain of Cpl-7 (IPR013168), SLaP: S-layer protein (IPR024968), AMIN: N-terminal nonamidase (IPR021731), SH3-1: sarcoma [src] homology 3 of type 1 (PF00018), SH3-2 (PF07653), SH3-3 (PF08239), SH3-4 (PF06347), SH3-5 (PF08460), SH3-6 (PF12913), SH3-7 (PF12914), SH3-8 (PF13457) and SH3-9 (PF14604)*.

f *Main phyla of the kingdom Bacteria and examples of some of the main bacterial genera (in brackets and in italics) with bacterial genomes encoding protein harboring the CWA catalytic domain as recorded in InterPro and Pfam databases*.

#### Cysteine Histidine-Dependent Amidohydrolases/Peptidases (CHAP)

The CHAP domain is around 120 amino acids long (IPR007921) with the unique feature to be potentially involved in two different CW cleavage activities (Bateman and Rawlings, 2003; Rigden et al., 2003). As a peptidase, it cleaves between D-alanine and the first glycine of the pentaglycine cross-bridge, but it can also act as an amidase by cleaving the chemical bond between MurNAc and L-alanine at the N-terminal of the stem peptide (Bateman and Rawlings, 2003; Rigden et al., 2003). Depending on the CHAP-proteins, some have been described as having only a peptidase activity (e.g., LysK), only amidase activity (e.g., Skl), or both (e.g., LytN) (Llull et al., 2006; Becker et al., 2009; Frankel and Schneewind, 2012). In phage proteins, the CHAP domain is mostly positioned in the N-terminal region, whereas in bacterial proteins it is systematically positioned at the C-terminal (Zou and Hou, 2010). The bacteriophage LysK was active on purified PG of a number of pathogenic strains including a wide range of staphylococci (O'flaherty et al., 2005; Becker et al., 2009). Resolution of the CHAP structure revealed a calcium-binding site, where calcium ion appeared to play a key role in the switch from active to inactive states (Gu et al., 2014; Sanz-Gaitero et al., 2014; Keary et al., 2016; Broendum et al., 2018). In CHAP SSP0609 from *S. saprophyticus*, a highly-conserved Cys-His-Glu-Asn tetrad relaying the active site was further evidenced (Rossi et al., 2009). While a set of hydrophobic residues forming a large cavity that would confer specificity to the binding site was lacking in the immediate vicinity of the active site, two Tyr aromatic residues likely play a role in substrate anchoring.

#### New Lipoprotein C/Protein of 60-kDa (NlpC/P60)

The NlpC/P60 domain is about 100 amino acids long (IPR000064) and proteins of this superfamily are ubiquitous papain-like cysteine peptidases involved in the catalysis of the *N*-acetylmuramate-L-alanine or D-γ-glutamyl-meso-diaminopimelate linkages (Anantharaman and Aravind, 2003). Four major families have been identified, namely (i) the P60-like family, (ii) the AcmB/LytN-like family, (iii) the YaeF/Poxvirus G6R family, and (iv) the lecithin retinol acyltransferase-like (LRAT-like) family (Anantharaman and Aravind, 2003). These two latter enzyme families contain a circularly permuted catalytic domain where the relative positions of the cysteine and histidine/polar residue of the conserved catalytic triad are swapped in the primary sequence (Anantharaman and Aravind, 2003). In *B. subtilis*, LytF and LytE, which belong to the P60-like family, break the linkage of m-diaminopimelic acid of the PG (Yu et al., 2016). Structure determination of NlpC/P60 domain in CwlT from *Clostridioides difficile*, previously known as *Clostridium difficile* or *Peptoclostridium difficile* (Yutin and Galperin, 2013; Lawson et al., 2016), revealed a conserved Cys-His-His-Tyr active site specific to tetrapeptide (Xu et al., 2014). Switch between catalysis in the closed state to the substrate entry or product release in the open state is likely regulated by a side chain above the catalytic Cys residue.

#### Peptidase M15 (PM15)

The PM15 domain is approximately 200 amino acids long (IPR000755) and can be further discriminated into four subfamilies, namely from PM15A to PM15D. In *Enterococcus faecium*, the structure of the D-Ala-D-Ala dipeptidase VanX has been solved and revealed a Zn-dependent catalytic mechanism (Bussiere et al., 1998). In contrast, the D,D-carboxypeptidase VanY is active against pentapeptide but lacks activity against dipeptides (Wright et al., 1992). The D,D-carboxypeptidase VanXYc from *Enterococcus gallinarum*, however, can further hydrolyse pentapeptides (Meziane-Cherif et al., 2014). Following structural and biochemical analyses, the molecular basis of this dual specificity was unraveled and appeared to result from the flexibility of a mobile cap region over the catalytic site, which allows the switch between di- and pentapeptide hydrolysis (Meziane-Cherif et al., 2014). Such a structural element would be lacking from VanY enzymes. While structure comparison revealed similarity in the catalytic sites of VanXY and VanX, differences were pinpointed at the D-Ala-D-Ala binding sites especially for a second cavity present in VanXY but with no equivalent in VanX.

#### Peptidase M23 (PM23)

The PM23 domain is around 100 amino acids long (IPR016047). Lysostaphin from *Staphylococcus simulans* is one of the best studied PM23. In its active form, lysostaphin cuts the peptide bond between the third and fourth glycine residues of the pentaglycine cross-link in the PG of *Staphylococcus* (Schneewind et al., 1995). The active enzyme shares structural similarity with Ale-1 and LytM (Sabala et al., 2014). In *Staphylococcus capitis*, however, the N-terminal repeats of the lysostaphin homolog Ale-1 are not processed post-translationally (Sugai et al., 1997; Lu et al., 2006). In *S. aureus*, LytM is produced as a latent CWH, where the occluding region and N-terminal domain need to be removed for its activation (Sabala et al., 2012). Following resolution of the enzyme structure (Odintsov et al., 2004), the importance of Zn^2+^ cation in the catalysis was confirmed, whereas the active site appeared to be located at the bottom of an extended, long and narrow groove, partially filled up by a loop, which would constitute the substrate-binding cleft (Firczuk et al., 2005; Grabowska et al., 2015). Beside the preferential glycine-glycine bonds, characterization of several PM23 suggested that they can also be less frequently active against alanine-glutamine bonds (Schindler and Schuhardt, 1964; Horsburgh et al., 2003a).

#### Peptidase M74 (PM74)

The PM74 domain is about 230 amino acids long (IPR005073). The PM74 D,D-endopeptidase MepA from *E. coli* cleaves the D-alanyl-meso-2,6-diamino-pimelyl amide bond in PG (Keck et al., 1990; Van Heijenoort, 2001). Structural investigations further revealed the enzymatic activity of MepA was Zn dependent and consequently sensitive to metal chelators (Keck et al., 1990; Van Heijenoort, 2001; Marcyjaniak et al., 2004).

#### Peptidase S11 (PS11)

The PS11 domain is approximately 235 amino acids long (IPR001967) and is systematically associated with another C-terminal domain (IPR012907), which is organized into a sandwich of two anti-parallel β-sheets. The function of this C-terminal domain is unknown, but it could mediate interaction with other CW synthesizing enzymes (Davies et al., 2001). The PS11 is involved in hydrolysis of the D-Ala-D-Ala bond and is present in several penicillin-binding proteins (PBPs) (Van Heijenoort, 2001; Macheboeuf et al., 2006). Investigation of the enzymatic mechanism stressed the importance of the Lys-Ser-Cys at the catalytic site (Fonze et al., 1999; Rhazi et al., 2003).

#### Peptidase M14 (PM14)

The PM14 domain is about 270 amino acids long (IPR005073). Together with PS13 and PS66 domains, little experimental work has been dedicated to PM14 domain. In *E. coli*, the PM14 MpaA hydrolyses the γ-D-glutamyl-diaminopimelic acid bond in the murein tripeptide L-alanyl-γ-D-glutamyl-*meso*-diaminopimelic acid (Uehara and Park, 2003). Structural analysis revealed the entrance and binding of the substrate into the active groove was mediated by a loop with critical Tyr-Asp residues (Ma et al., 2017).

#### Peptidase S13 (PS13)

The PS13 domain is around 430 amino acids long (IPR000667). The PS13 PBP4 from *E. coli* exhibits both D,D-carboxypeptidase and D,D-endopeptidase activities (Van Heijenoort, 2011).

#### Peptidase S66 (PS66)

The PS66 domain is about 315 amino acids long (IPR003507). The PS66 LdcA from *E. coli* is a L,D-carboxypeptidase releasing the terminal D-alanine residue from the tetrapeptide L-Ala-γ-D-Glu-*meso*-A_2_pm-D-Ala (Templin et al., 1999).

#### Transpeptidase (TP)

The TP domain is approximately 120 amino acids long (IPR005490). The L,D-transpeptidase LdtMt2 from *Mycobacterium tuberculosis* harbors a TP domain and has been the subject of intense structural investigations, especially for the study of drugs blocking the enzyme activity through PG cross-linking (Kim et al., 2013; Li et al., 2013; Bianchet et al., 2017; Kumar et al., 2017; Gokulan et al., 2018). In *E. coli*, YbiS, ErfK, YcfS, YcbB, and YnhG also harbor a TP domain but exhibit both L,D-transpeptidase and L,D-carboxypeptidase activities (Magnet et al., 2008; Van Heijenoort, 2011). These five enzymes possess a sole Cys residue essential for activity. While YbiS, ErfK and YcfS enable to cross-link lipoprotein to PG, YcbB and YnhG catalyse the cross-linking of mDAP to form direct meso-diaminopimelate (DAP-DAP, also called linkage of 3-3 type) (Sanders and Pavelka, 2013). Actually, the majority of the cross-linking of the stem peptides involves penicillin-binding proteins (PBPs) with D,D-transpeptidase activity (IPR001460) and corresponds to the 4-3 type, where a D-alanine residue at the fourth position of the peptide stem links at a mDAP at the third position of an adjacent peptide stem (Glauner et al., 1988; Arbeloa et al., 2004). While Csd6 from *Helicobacter pylori* clearly possesses a TP domain, it would not function as a L,D-transpeptidase at all but as a L,D-carboxypeptidase (Kim et al., 2015). This stresses the need for further investigations to gain insight in the diversity of the TP domain, most certainly into subfamilies and probably a redefinition of this domain family.

### The Diversity of the Modular Architecture of Cell Wall Hydrolases

Besides the different catalytic domains involved in PG hydrolysis and reviewed here above, numerous CWHs have CW binding domains (Table 1 and Figures 3–5). In bacteria, several conserved domains have been reported as involved in covalent or non-covalent binding of proteins to CW components (Desvaux et al., 2006, 2018; Tables 1–3). The optimal type and number of anchoring domains required for maximum binding differs from one protein to another (Visweswaran et al., 2014). As a general rule, these domains allow binding of the enzymes to the CW at an adequate concentration and properly position the active site toward the PG substrate site for formation of an efficient enzyme-substrate complex (Steen et al., 2005; Bosma et al., 2006; Shao et al., 2012). Because of close spatial proximity, the released hydrolysed products can be readily and efficiently transported into the cell for recycling (Ozdemir et al., 2012).

LysM (IPR018392), one of the most frequent CW binding domains found in CWHs, is found at the N- or C-terminal of a protein (Buist et al., 2008). The number of domains found in a CWH varies from one to 12, and these domains are generally separated by serine-threonine-asparagine rich intervening sequences (Bateman and Bycroft, 2000; Visweswaran et al., 2014). When present, PGB1 (IPR002477) is usually found in a single copy at the N- or C-terminal; although up to nine repeats can be found in some proteins (Desvaux et al., 2006). The SLH (S-layer homology domain; IPR001119) is generally found at the C-terminal of CWHs. In the NALAA-3 AmiC from *E. coli*, the AMIN (N-terminal nonamidase; IPR021731) domain allows proper localization of the enzyme at the division site through binding to the PG (Rocaboy et al., 2013).

**Figure 6:**
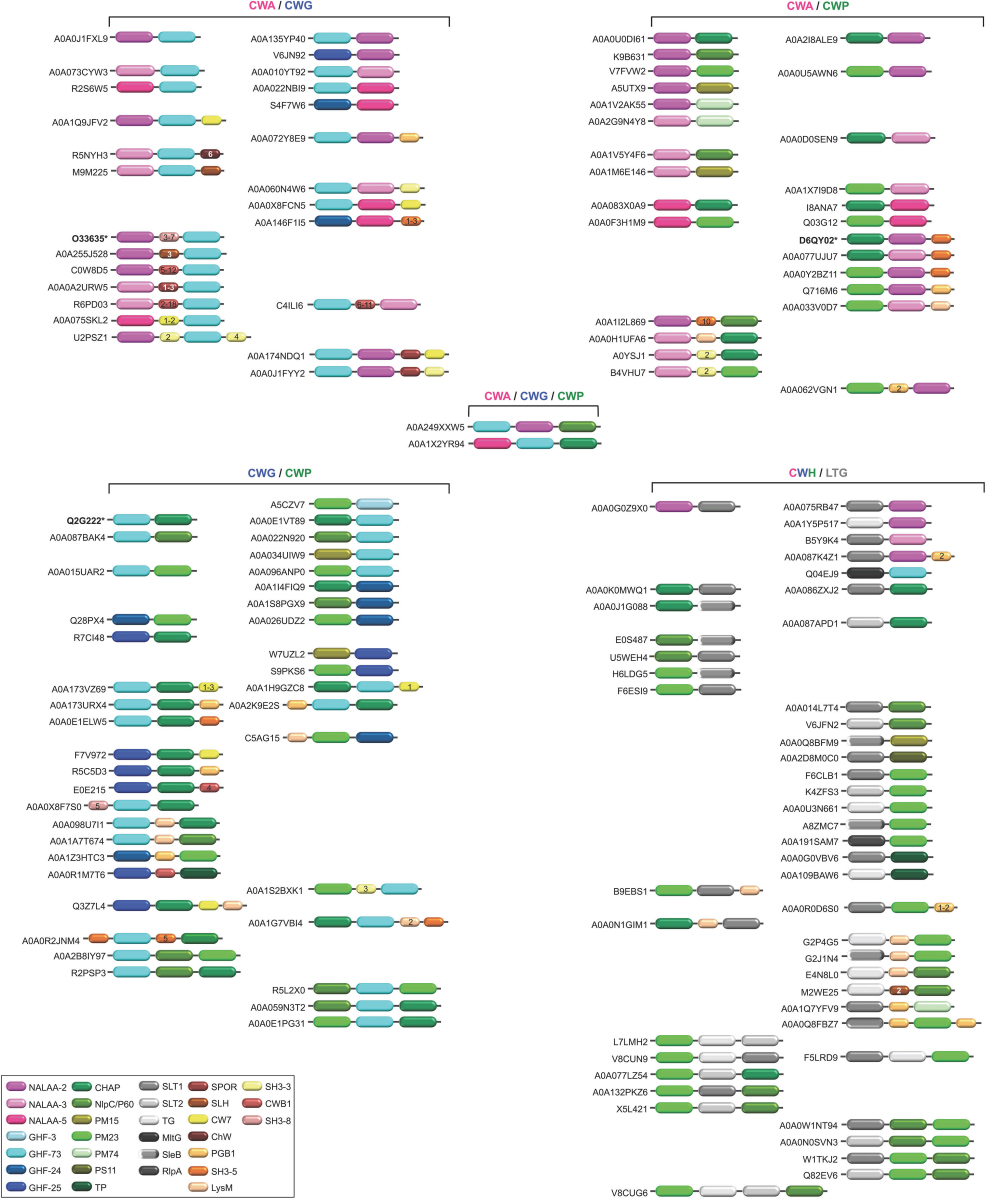
Schematic representation of the diversity of modular architectures of multifunctional CWHs, i.e., acting as CWA-CWG, CWA-CWP, CWG-CWP, CWA-CWG-CWP, or CWH-LTG. For each modular organization, a UniProt identifier is provided as a representative. Bold identifier indicates that at least one representative protein has been functionally characterized (including the given identifier). Asterisk indicates that at least one representative has been structurally characterized (including the given identifier).

In CWHs, nine different types of SH3 (sarcoma [src] homology 3; IPR003646) domains can be found, from SH3 of type 1 (SH3-1) to SH3-9 (Tables 1–3). The SH3 domains can be found in the N- or C-terminal region of a CWH (Figures 3–6). This domain contains five β-strands forming two orthogonal anti-parallel β-sheets of two and three β-strands each (D'aquino and Ringe, 2003; Desvaux et al., 2006, 2018). In PlyTW (phage Twort endolysin), it has been demonstrated that loss of SH3 domain results in an ~10-fold reduction in enzymatic activity (Becker et al., 2015). Deletion analysis of SpAE (staphylococcal phage 2638A endolysin) indicates that the NALAA-2 domain confers most of the lytic activity and requires the full SH3 domain for maximal activity (Abaev et al., 2013). Actually, loops from SH3 domains can dock into the ends of the active site groove of the catalytic site, remodel the substrate binding site and modulate substrate specificity (Xu et al., 2015).

Actually, more than one CW binding domain can be found in some CWHs (Figures 3–6). While it can be hypothesized that such combinations are beneficial for catalysis, much remains to be learned about their effect on PG hydrolysis. Studies have yet to be made of the structure-function relationships and structural constraints regarding the large varieties of combinations between different catalytic domains and/or CW binding domains (Alcorlo et al., 2017; Broendum et al., 2018).

The different catalytic domains reviewed above can be found individually or in combination in various CWHs (Tables 1–3 and Figure 6). CWHs with only one catalytic domain do not necessarily have a CW binding domain but very often CWHs harboring an additional catalytic domain also have a CW binding domain (Visweswaran et al., 2014). Numerous different combinations of CWA, CWG, and CWP domains can be found, although the diversity of associations of CWA or CWG domains with CWP domains or the simultaneous presence of CWA, CWG, and CWP domains in a single CWH is rare (Figure 6).

Most CWAs exhibit only one catalytic domain, either NALAA-2, NALAA-3, or NALAA-5 (Figure 3); no CWA combining two types of amidase catalytic domain has been reported to date (Table 1). However, in some CWHs, these CWA domains can be found in association with either a CWG domain, especially GHF-73, or a peptidase domain, such as PM23 (Albrecht et al., 2012) (Figure 6). As in CwlA from *B. subtilis* (Foster, 1993), NAALA-2 can be found in association with GHF-25. The major CWHs AtlA from *S. aureus* and AtlE from *S*. *epidermidis* are synthesized as propeptides with a NALAA-2 domain together with a GHF-73 domain (Oshida et al., 1995; Heilmann et al., 1997; Albrecht et al., 2012). The propeptide is cleaved off by an extracellular protease generating two extracellular CWHs, namely a 51 kDa endo-β-N-acetylglucosaminidase and a 62 kDa N-acetylmuramoyl-L-alanine amidase, which are independently involved in the partitioning of daughter cells after cell division (Yamada et al., 1996; Götz et al., 2014). Regarding peptidase domains associated with an amidase domain, SpAE has a N-terminal PM23 domain together with a central NALAA-2 domain (Abaev et al., 2013). Deletion analysis indicates that the NALAA-2 domain confers most of the lytic activity (Abaev et al., 2013). In contrast, in LytA from *S. aureus* where a N-terminal CHAP domain is associated with a central NALAA-2 domain, the lytic activity is prominently conferred by the CHAP domain (Havarstein et al., 2006). By generating a truncation in the homologous CWH LysK (from bacteriophage K in *Lactococcus lactis*), where only the first 165 amino acids are kept, an activity twofold higher than that of the native protein was observed (Rigden et al., 2003; Horgan et al., 2009). The endolysin PlyTW from bacteriophage Twort has a similar architecture to LytA and LysK, and its CHAP domain alone is sufficient and necessary for cell lysis but the NALAA-2 domain alone is insufficient (Becker et al., 2015).

**Figure 7:**
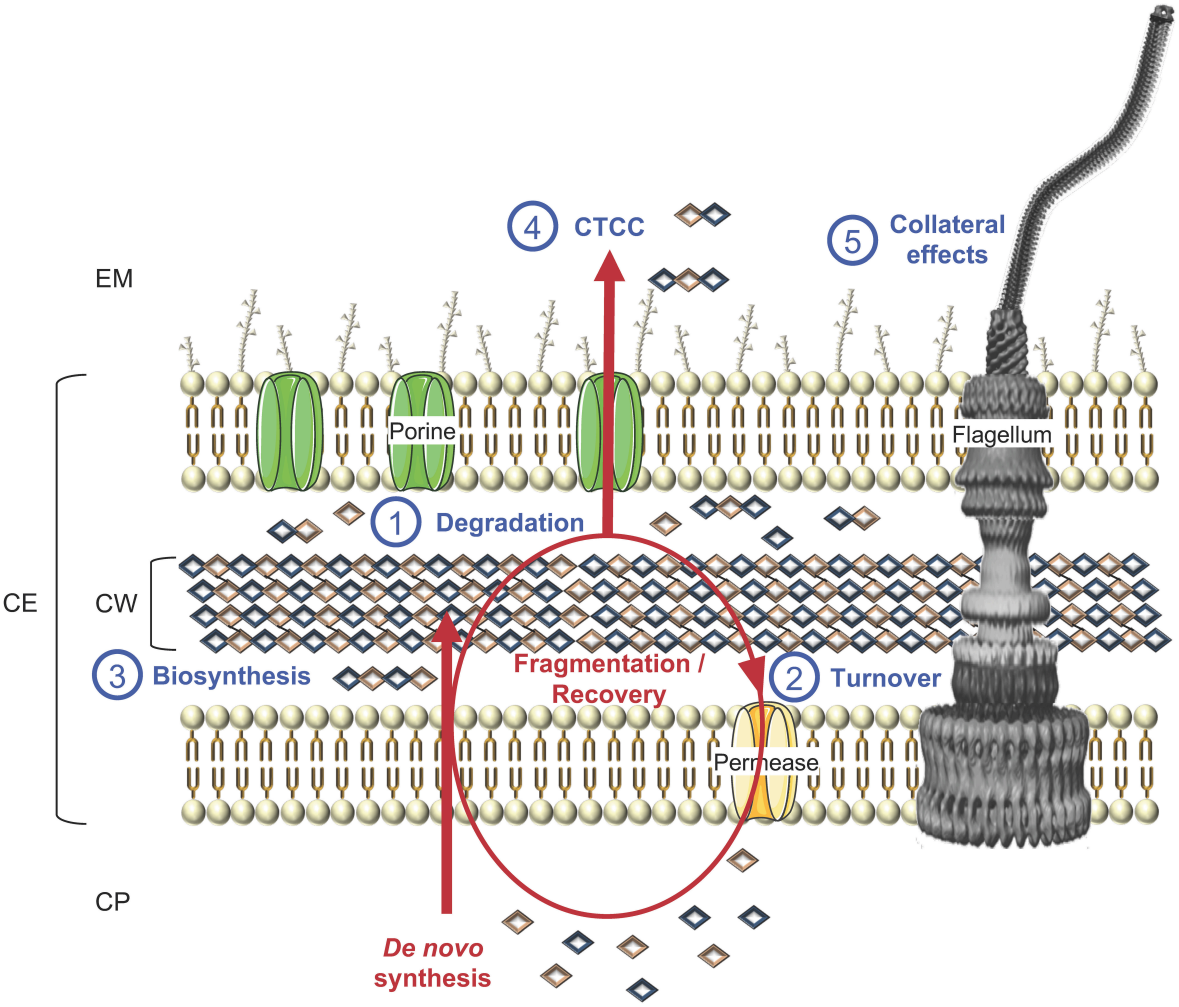
Schematic representation of cell wall biosynthesis and recycling, exemplified in a LPS-diderm bacterial cell. Besides (1) degradation, which can lead to cell lysis, CWHs also participate to cell wall rearrangement and other key physiological functions, namely (2) turnover of cell wall material through their recycling to (3) biosynthesis, as well as (4) cell-to-cell communication (CTCC) since some released peptidoglycan fragments can act as signaling molecules or have (5) side effects on final protein subcellular localization, e.g., flagella, with consequences on motility, bacterial adhesion, biofilm formation, protein secretion, conjugation, virulence and/or immune response. CP, cytoplasm; CE, cell envelope; CW, cell wall; EM, extracellular milieu.

CWG can either display one glycosidase domain alone or in association with a peptidase domain (Figure 6). While only the GHF-25 and GHF-73 glycosidase domains can be found in association with an amidase domain, several peptidase domains can be associated with glycosidase. Among the four CWHs from *L. lactis*, AcmB (N-acetylmuramidase type B) harbors a central GHF-73 domain and a C-terminal CHAP domain (Huard et al., 2003, 2004; Visweswaran et al., 2013). Qualitatively, a truncated version of the enzyme lacking the CHAP domain seems less efficient than the enzyme with the dual catalytic sites for CW hydrolysis (Huard et al., 2003). Considering the low p*I* (5.03) of the mature protein, and that a hydrolytic activity can only be detected at acidic pH in renaturing conditions, this suggests that interaction with the negatively charged CW is favored by a positive or neutral charge of the protein. Unlike CWAs, some CWGs and CWPs can combine two different types of glycosidase or peptidase catalytic domains, e.g., PS11 and PS13 (Tables 2, 3 and Figure 7). However, the functional characterization and benefit for enzymes with such a modular architecture has not been yet investigated. With regards the wealth of diverse combinations, much remains to be learned about the inhibiting and synergistic effects of the interactions between amidase, glucosidase and peptidase domains in a monopolypeptidic CWH.

Of interest, some CWHs can also harbor additional catalytic domains involved in CW degradation but unrelated to hydrolases, namely the LTGs (Figure 6). In fact, the molecular reaction mechanism for LTGs does not involve water (Höltje et al., 1975). Based on conserved sequence motifs (Herlihey and Clarke, 2017), LTGs are discriminated between SLT1 (soluble lytic murein transglycosylase of type 1; IPR008258), SLT2 (IPR031304), SleB (Spore cortex-lytic enzyme of *Bacillus*; IPR011105), TG (transglycosylase; IPR010618), MltA (membrane-bound lytic transglycosylase of type A; IPR005300), MltG (previously known as YceG; IPR003770) and RlpA (rare lipoprotein A; IPR034718) domains (Blackburn and Clarke, 2001; Scheurwater et al., 2008; Vollmer et al., 2008b; Li et al., 2012). Of note, LysG (IPR023346; PF13702) constitutes an additional LTG domain but further biochemical characterisations are still required to confirm this (Xu et al., 2014). While MtlA catalytic domain could never be found associated with any CWH domain, SLT1 can be found together with some CWA domains, namely NALAA-2 or NALAA-3, and/or some CWP domains, namely CHAP, NlpC/P60, PM23, and TP (Table 1). SLT2, TG and RLpA could only be found in some CWPs. Among CWGs, only MltG could be found in association with GHF-73.

## Cell Wall Hydrolases From Cell Division, Through Cell Wall Rearrangement, to Recycling and Cell Lysis, up to Collateral Effects

CWHs are an essential part of bacterial cell physiology. These ubiquitous enzymes have important roles in cell division and CW rearrangement in creating space within the PG to accommodate supramolecular structures for the secretion and assembly of flagella or pili (Scheurwater et al., 2008). PG recycling is tightly coordinated with its biosynthesis in a carefully controlled manner to prevent the loss of CW integrity, which would lead to cell lysis and bacterial death (Figure 7). Beyond bacterial growth and cell lysis, CWH activity can have side effects on a wide range of physiological functions from bacterial adhesion, biofilm formation, protein secretion, conjugation, virulence and immune response.

### Cell Division and Cell Wall Rearrangement

In the course of bacterial cell growth, PG is biosynthesised through well-known anabolic pathways, including (i) the synthesis of MurNAc pentapeptide precursor (Barreteau et al., 2008; Sobhanifar et al., 2013), (ii) the attachment of the precursor to the membrane anchored lipid carrier completing the formation of the PG precursor lipid II (Bouhss et al., 2008), and (iii) the polymerisation of the PG by PBPs, glycosyltransferases, L,D- and D,D-transpeptidases after translocation of lipid II across the cytoplasmic membrane, (Sauvage et al., 2008; Typas et al., 2012). The majority of low molecular weight PBPs acts as D,D-carboxypeptidases that help to control the extent of cross-linking through hydrolysis of the carboxy D-Ala-D-Ala peptide bond of a stem peptide (Scheffers and Pinho, 2005). D,D-carboxypeptidases remove terminal D-Ala residues at position 5 of pentapeptides in the PG and regulate PG synthesis (Egan et al., 2015). For example, a *S. pneumoniae* mutant lacking the D,D-carboxypeptidase PBP3 forms aberrant septa and has a thickened CW, whereas an *E. coli* mutant lacking PBP5 has bent or even branched cell shapes (Nelson and Young, 2001; Vollmer et al., 2008b). PG growth or elongation requires the cleavage of covalent bonds by hydrolases to allow the newly attached material to be inserted in the layer without increasing its thickness (Typas et al., 2012; Vollmer, 2012). In *E. coli*, the three CWPs, Spr, YdhO (belonging to NlpC/P60) and YebA (belonging to PM23), are collectively required for cell growth and PG incorporation (Singh et al., 2012). The depletion of these endopeptidases results in growth arrest and lysis in *E. coli* and prevents further incorporation of new PG (Vollmer, 2012).

In *B. subtilis*, the disruption of two D,L-endopeptidase genes *cwlO* and *lytE* (belonging to NlpC/P60) is lethal (Bisicchia et al., 2007). Inactivation of the LytF CWP harboring a Nlpc/P60 domain lead to slightly filamentous cells, whereas the *lytF lytE* double mutant forms extra-long chains (Ohnishi et al., 1999). In *S. aureus*, the Sle1 CWP with a CHAP is required for cell separation (Kajimura et al., 2005). However, it is not yet known how the CWHs required for cell growth are positioned and regulated and how their depletion results in a halt in PG incorporation. In accordance with the “make-before-break” strategy (Koch and Doyle, 1985), a new three-for-one growth mechanism has been proposed (Holtje, 1996, 1998), namely for every three new PG strands inserted in the CW, one old strand is replaced. The existence of multienzyme complexes in *E. coli* was proposed to combine the activities of PG synthases (transpeptidases and transglycosidases) and PG hydrolases (Holtje, 1996, 1998; Scheffers and Pinho, 2005). Affinity chromatography with immobilized lytic enzymes such as MltA, Slt70 or MltB has indeed identified interactions with PG synthases (Vollmer et al., 2008a).

PG cleavage is also required for reductive cell division and cell separation. *E. coli* has 13 periplasmic CWHs that can collectively cleave almost any glycoside, peptide or amide bond (Vollmer et al., 2008b). No single hydrolase gene knockout prevents growth of *E. coli*, probably owing to high redundancy. In fact, multiple hydrolases genes have to be deleted to form chains of non-separated cells (Vollmer et al., 2008b). CWAs have a prominent role in septum cleavage, in comparison with that of glycosidases and peptidases (Heidrich et al., 2001, 2002; Priyadarshini et al., 2006; Typas et al., 2012). The AmiA, AmiB, and AmiC CWAs play an important role in releasing daughter cells after cell division (Heidrich et al., 2001). AmiC appears to be the principal septum-cleaving enzyme in *E. coli*. Indeed, mutants inactivated in AmiC separate poorly, with about 30% of the population existing as chains of 3–6 unseparated cells, vs. 5–10% of the population in chains of 3–4 cells when only *amiA* is deleted, and no chaining effect upon deletion of *amiB* (Heidrich et al., 2001). In a mutant lacking AmiA, AmiB and AmiC CWAs, more than 90% of the cells exist as unseparated chains from 6 to 24 cells long (Priyadarshini et al., 2006). The cumulative effect of deleting all CWAs and CWPs as well as the Slt70 CWG is even more severe (Priyadarshini et al., 2006). The endopeptidases of the M23-LytM family are also implicated in the septation of *E. coli* cells (Typas et al., 2012)*. E. coli* has four LytM paralogues, namely EnvC, NlpD, YgeR, and YebA, and their inactivation results in default in cell separation (Typas et al., 2012). In CW-monoderm bacteria, cleavage of the septum occurs simultaneously with cell division (Vollmer et al., 2008a). Several hydrolases contribute to this step, for example, an *atl* mutant of *S. aureus* and a *lytB* mutant of *S. pneumoniae* form clusters of non-separated cells (Vollmer et al., 2008a). Both hydrolases are localized at the sites of cell division.

In addition, CWHs can interact with each other and modulate their catalytic activity. In *E. coli*, the cell division proteins FtsN and FtsEX activate paralogs of LytM endopeptidases, namely EnvC and NlpD respectively, which further activate three amidases involved in the splitting of septum (Uehara et al., 2010; Typas et al., 2012); NlpD activates AmiC and EnVC activates AmiA and AmiB. In *B. subtilis*, FtsEX has also been shown to regulate the activity of CwlO (Dominguez-Cuevas et al., 2013; Meisner et al., 2013). Similarly, in *S. pneumoniae*, FtsEX activates the hydrolase PcsB (Sham et al., 2011). This hydrolase is essentially controlled by the sensor regulator YycFG (Ng et al., 2003). The allosteric activation of *E. coli* amidase AmiB requires structural modification of the active site for substrate binding, and this mechanism seems to be conserved in amidases cleaving the septum (Yang et al., 2012). Similar activation by one or more protein(s) could also be applied to the *E. coli* D,D-endopeptidases Spr, YdhO, and YebA, which have low *in vitro* activity against PG (Singh et al., 2012). In the case of *B. subtilis*, the expression of the muramidase YocH and of the endopeptidases CwlO, CwlF and LytE is controlled by the YycFG two-component system, which plays a key role in synchronizing CW metabolism and division (Bisicchia et al., 2007). The PG lipid II precursor or the process of its incorporation could be the signal sensed by YycFG (Bisicchia et al., 2007). In LPS-diderm bacteria, CWH activity is controlled by incorporation into multi enzyme complexes that span the periplasm, extending from inner membrane-anchored synthases to the hydrolases, some of which are associated with the outer membrane (Typas et al., 2012). Hydrolases in the complex may be localized at sites of PG synthesis, preventing them from hydrolysing PG elsewhere (Typas et al., 2012). In CW-monoderm bacteria, a direct interaction between PG synthases and hydrolases is generally not possible because they are physically separated; the synthases act in the inner face and the hydrolases on the outer layers (Carballido-Lopez et al., 2006). As already mentioned in *B. subtilis*, though, CwlO is activated by the membrane protein complex FtsEX, it has been suggested that its activity is restricted to the inner part of the PG layers (Dominguez-Cuevas et al., 2013). In this species, PG synthesis is controlled by the actin-like cytoplasmic protein MreBH, which forms filaments at the cytoplasmic membrane and directs the PG synthesis by co-localization of the synthases and the hydrolases (Carballido-Lopez et al., 2006). While colocalisation of synthases and hydrolases was proposed as model for the control of PG synthesis by the MreB cytoskeleton, later work in the field rather suggests that the localization of PG synthases and hydrolases is uncoupled (Dominguez-Cuevas et al., 2013; Meisner et al., 2013).

LTGs are involved in the assembly of large *trans*-envelope structures such as the secretion system of type II (T2SS), T3SS and T4SS, as well as surface organelles, including type 4 pili (T4P) and flagella (Koraimann, 2003; Zahrl et al., 2005; Scheurwater and Burrows, 2011; Herlihey and Clarke, 2017). They are required to enlarge gaps locally in the PG to allow the efficient assembly and anchoring of transport complexes in the cell envelope (Koraimann, 2003). For examples, VirB1 is involved in the assembly of the T4SS (Hoppner et al., 2005), FlgJ in the flagellum (Nambu et al., 1999; Hirano et al., 2001), and EtgA in the T3SS (Garcia-Gomez et al., 2011). While PilT is considered as involved in T2SS assembly, including T4P (Koraimann, 2003), its role remains to be ascertained. In *B. subtilis*, LytC and LytD CWHs have mutually compensatory roles in CW turnover and cell separation as well as motility (Horsburgh et al., 2003b). Besides cellular lysis, the AcmA CWG is involved in cell remodeling in *L. lactis* (Huard et al., 2004; Steen et al., 2005). In *Salmonella* Typhymurium, FlgJ (flagellar protein J) exhibits a GHF-73, whose CWG activity is essential for flagella assembly as it degrades the PG locally to enable the formation of the rod structure in the periplasmic space (Nambu et al., 1999).

### Recycling and Cell Lysis

It has been established that a turnover of around 45% of the CW material occurs over one generation in bacteria (Goodell, 1985). In CW-monoderm bacteria, where PG generally accounts for more than 20% of the cell mass, vs. 2% in LPS-diderm bacteria, this would result in a significant loss of resources (Johnson et al., 2013). By providing the building blocks for CW biosynthesis, recycling is an energy saver mechanism for bacterial growth (Figure 7). Besides, when the bacterial cells are faced with a sudden loss of carbon, recycling of CW material would allow bacterial survival by completing a last round of cell division before growth arrest (Park and Uehara, 2008). While few data are available for CW-monoderm bacteria, *E. coli* has been widely used to investigate the processes of CW recycling (Park and Uehara, 2008). The major pathway involves the uptake of the anhydromuropeptides released in the periplasm from PG hydrolysis by the transporter AmpG specific for GlcNAc-1,6-anhMurNAc and GlcNAc-1,6-anhMurNAc-peptides (Park and Uehara, 2008). These anhydromuropeptides are further degraded in the cytoplasm by the N-acetylglucosaminidase NagZ, the anhMurNac-Lala amidase AmpD and the L,D-carboxypeptidase LdcA, and then join the PG synthesis pathway (Holtje et al., 1994; Templin et al., 1999; Cheng et al., 2000; Reith and Mayer, 2011; Johnson et al., 2013). While AmpD and AmpG are absent from CW-monoderm bacteria, CWGs of the lysozyme family play a more prominent role than N-acetylglucosaminidase or LTGs (Reith and Mayer, 2011). Rather than anhydromuropeptides, lysozymes release MurNAc-containing muropeptides. In addition, hydrolysis of the stem peptides by amidases and peptidases plays a crucial role in PG degradation in CW-monoderm bacteria (Reith and Mayer, 2011). It has been shown that the CW of *S. aureus* must be pre-digested first with amidase before it becomes a good substrate for the glucosaminidase (Götz et al., 2014). Muropeptide cleavage occurs in the extracellular space of the CW compartment. Uptake of CW amino sugars will occur through the phosphotransferase system PTS permeases (NagP, MurP) and uptake of CW peptides through Mpp/Opp-like ABC transporters (Johnson et al., 2013). In *S. aureus* and *B. subtilis*, respectively, about 5 and 10% of the MurNac of the CW is recycled per generation (Borisova et al., 2016).

Autolysis can be induced by the inhibition of PG synthesis in growing cells (Vollmer et al., 2008b; Van Heijenoort, 2011). In this case, the autolysis in *E. coli* is not due to any specific induction of hydrolases but rather to an uncoupling between synthesis and degradation owing to an absence of control of the turnover CWHs (Van Heijenoort, 2011). Treatment of growing cells of *E. coli* with penicillin reduces the rate of PG synthesis and increases net lysis; a correlation has been established between induction of autolysis and inhibition of synthesis and degradation of PG by CWHs (Vollmer et al., 2008b). Autolysis of *E. coli* is low for non-growing cells and this can be attributed to PBP7 endopeptidase involvement in D-Ala-D-*meso*-A2pm cross-linkage, which contributes to structural changes of PG (Van Heijenoort, 2011). The activity of DdpX CWP exhibiting PM15 is considered as potentially lethal and owes to the hydrolysis of the D-Ala-D-Ala bond (Lessard et al., 1998). Overproduction of PBP5 CWP encoded by *dacA* and harboring a PS11 domain appeared to be lethal, causing *E. coli* cells to grow as spherically before lysing (Markiewicz et al., 1982). PBP5 regulates the availability of pentapeptide subunits for the formation of the cross-linkages by transpeptidation (Broome-Smith et al., 1988; Potluri et al., 2010; Van Heijenoort, 2011).

The myxobacteria, which are predatory bacteria, produce extracellular lytic agents including CWHs such as lysozyme and endo-β-N-acetylglucosaminidase (Bourgerie et al., 1994). Similarly, *Pseudomonas aeruginosa* and other LPS-diderm bacteria deliver NlpC/P60 type endopeptidases via T4SS into the periplasm of adjacent bacterial cells causing their lysis (Chou et al., 2012). The production of bacteriolytic exo-enzymes is also a property of many species of actinomycetes, in particular streptomycetes (Vollmer et al., 2008b). Lysis phenomena have been observed in the sporulation stage of myxobacteria and *Bacillus*, and will result in the release of nutrients (Vollmer et al., 2008b). In *B. subtilis*, the CwlB and CwlC CWAs with a NALAA-3 domain are present in large amounts at the time of mother cell lysis (Smith and Foster, 1995). While single inactivation of *cwlB* or *cwlC* does not affect mother cell lysis, it was found to be blocked upon double gene knock-out (Nugroho et al., 1999). The CwlH CWA with a NALAA-2 domain was shown to be required for mother cell lysis and acts in a compensatory manner with CwlC (Nugroho et al., 1999; Yang et al., 2013). As CwlC, LytC is expressed only late in sporulation but is also involved in mother lysis (Smith and Foster, 1995).

Competent cells of *S. pneumoniae* are able to lyse non-competent cells during co-cultivation, a phenomenon termed allolysis (Guiral et al., 2005). The mechanism involves the two-peptide bacteriocin CibAB and its immunity factor CibC, as well as the LytA, LytC, and CbpD CWHs (Guiral et al., 2005). The bacteriocin activates the hydrolases, which disrupt the CW of the non-competent cells. Regulated bacterial death and lysis have been characterized in studies on the control of the lytic cycle during bacteriophage infection (Rice and Bayles, 2008). The mechanism controlling the bacteriophage-induced lysis involves a holin and a cognate CWH (Desvaux, 2012). In a first mechanism, upon activation of the holin, the membrane becomes leaky and allows the escape of the CWH to the PG and thus its degradation. The second mechanism involves bacteriophages-encoded CWH containing signal arrest release (SAR) domains (Rice and Bayles, 2008). This SAR-type hydrolase is transported in a Sec-dependant manner and anchors in the outer face of the membrane in an inactive form until the holin releases it.

CWHs must be highly regulated to prevent cell lysis. This regulation occurs at both the transcriptional and post-translational levels. In *B. subtilis*, the transcription of the two hydrolases LytT and LytD is regulated by the sigma factor SigD, whereas LytE is regulated by SigA, SigH, and SigI (Serizawa et al., 2004). In *S. aureus*, perturbation of CW synthesis resulted in the modulation of the expression of the major CWH Atl by the two-component regulation system VraSR (vancomycin resistance-associated sensor/regulator) (Kuroda et al., 2003). In *S. aureus*, another two-component system LytSR controls the rate of autolysis by regulating the expression of *lrgAB* encoding an antiholin that interacts with holin to prevent cell lysis (Rice and Bayles, 2008). The interaction between the hydrolases and the CW is crucial for their activity, and their subcellular localization regulated at the post-translational level reflects their targeting mechanisms (Vollmer et al., 2008b). Furthermore, most of these hydrolases have a binding domain for PG or other CW components that greatly enhances their enzymatic activity. Proteolytic processing of CWHs is common both for their activity and their stability in response to different environments. For example, the hydrolase Atl of *S. aureus* is produced as a proenzyme that undergoes proteolysis resulting in mature cell-surface and extracellular glucosamidase and amidase (Komatsuzawa et al., 1997). In LytC from *S. pneumoniae*, conformational changes in the GHF-25 domain play a key role in the control of the enzymatic activity to avoid self-lysis during bacterial growth and division (Perez-Dorado et al., 2010).

Finally, the activity of the hydrolases is regulated by their environment, such as the presence or not of other CW polymers, as illustrated by the teichoic acids that modify the activity of the amidase in *B. subtilis* or the growth at acidic pH that inhibits lysis (Vollmer et al., 2008b).

### Collateral Physiological Effects

As reviewed above, the primary role of CWHs is the degradation of PG. The digestion can occur in a sparing manner for cleavage of the septum during cell division or spore maturation, enlargement of the sacculus, and assembly of supramolecular structures such as secretion systems, pili, or flagella (Vollmer et al., 2008b). The degradation can be more drastic in the case of the cell lysis, whether autolysis, allolysis, or exolysis (that is lysis of non-sibling prey cells). As a result of these CWH activities, several collateral effects of significance to bacterial physiology can further occur (Wyckoff et al., 2012). The expression of CWHs can affect the assembly of the supramolecular structure and indirectly modulate their affiliated function. For flagella, motility can be indirectly mitigated by FlgJ (Nambu et al., 1999), which could subsequently affect chemiotactism or surface colonization. By disturbing the assembly of pili, some CWHs could have side effects on bacterial aggregation, adhesion, biofilm formation, twitching motility or conjugation. For the secretion systems, some CWHs could incidentally modify the transport of proteins localized at the membrane, CW, periplasm, extracellular milieu or even injected into a host cell. Depending on the secreted proteins, a wide range of side effects could occur from substrate transport and cell adhesion to bacterial virulence.

In *L. monocytogenes*, CwhA (cell wall hydrolase A) was originally considered as a virulence factor *per se*, named Iap (invasion-associated protein) or P60 (protein of 60 KDa) at the time. Actually, it appeared to be primarily a CWH inducing septation default and consequently mislocalisation of key cell-surface virulence factors, namely the internalin InlA and the actin polymerisation factor ActA (Pilgrim et al., 2003; Desvaux and Hebraud, 2006). In addition, reduced secretion of both the CWHs CwhA and MurA via the SecA2 export pathway promotes extensive cell aggregation and sedimentation, as well as cell elongation inducing the formation of low-adherent filamentous biofilm (Machata et al., 2005; Renier et al., 2011, 2014). Regarding sessile development, the CWH activities can alter the global charge of the bacterial cell surface and/or modify the exposure of adhesin, which in turn modifies the bacterial aggregation/adhesion properties and/or biofilm formation abilities, as shown for AcmA from *L. lactis* (Mercier et al., 2002).

Besides recycling for CW biosynthesis, PG fragments released by the CWH activity could have messenger functions and act as signaling molecules (Boudreau et al., 2012) (Figure 7). In *E. coli*, muropeptides were shown to induce β-lactamase at the transcriptional level *via* the regulator AmpR (Jacobs et al., 1997). PG fragments could also initiate regrowth of dormant cells in the viable but nonculturable state (Keep et al., 2006). In the course of infection, these fragments can have proinflammatory activity and are recognized by PGRPs (peptidoglycan recognition proteins), which activate the immune response, especially the Toll or immune deficiency signal transduction pathways, or induce a proteolytic cascade generating antimicrobial compounds that induce phagocytosis or hydrolysis (Dziarski, 2003; Dziarski and Gupta, 2006; Markiewicz and Popowska, 2011). In *Neisseria gonorrhoeae* for instance, it is known for some times that PG fragments are cytotoxic to human ciliated Fallopian tube cells by causing their death and sloughing (Melly et al., 1984; Dillard, 2014). Besides, PG fragments from the neisserial CW influence the host innate immune response as they recognize and activate the NOD1 and NOD2 receptors (Mavrogiorgos et al., 2014; Knilans et al., 2017).

## Concluding Remarks

While the biochemical composition of the bacterial CW is now well-known, its supramolecular organization and the interaction of the different constituents still require further studies. Biophysical investigations with state-of-the-art approaches have been performed on a handful of model bacteria, namely *B. subtilis, S. aureus*, or *S. pneumoniae* for CW-monoderm bacteria but quite restricted to *E. coli* for LPS-diderm bacteria (Vollmer et al., 2008a; Turner et al., 2010; Beaussart et al., 2014). Beyond the simplistic dichotomy of Gram-positive vs. Gram-negative bacteria, there is a need to encompass the full biodiversity of the bacterial kingdom considering the divergent molecular compositions and structural arrangements of the CW. As presented in this review, this can be more effectively embraced by considering the trichotomy of CW-monoderm, LPS-diderm and myco-diderm bacteria.

Rather than the designations of autolysin, endolysin and exolysin, which are confusing, ambiguous and quite often used misleadingly in the scientific literature, this review stresses the relevance of using instead the global term CWH (or PGH) and the more specific designations of CWA, CWG, and CWP, for which conserved motifs and/or three-dimensional structures are clearly established. Besides, the combination of these latter terms, such as CWA-CWG or CWA-CWG-CWP, better reflect the modular organization of the numerous CWHs exhibiting multiple catalytic sites. This comprehensive review of the diversity of conserved domains exhibiting CWH catalytic activity will help scientists in the field to describe the enzymes at hand. While the association of different catalytic properties generally results in synergistic effects, the CW binding motifs that can be found along the monopolypeptide, generally improve the efficiency of the enzymatic activity (Shoseyov et al., 2006). While some CWHs attracted a lot of interest very early, e.g. the lysostaphin (Schindler and Schuhardt, 1964), this review also stresses numerous domains that have yet to be fully characterized and that only a handful of investigations have been dedicated to the biochemical, catalytic, molecular, structural and/or physiological characterization of enzymes with multiple catalytic sites and/or CW binding domains (Figures 3–6). In other words, the synergistic and processive effects of such combinations of domains have yet to be elucidated. Of note and besides LTG domains, some CWHs can exhibit additional catalytic domains unrelated to CW degradation or CW biosynthesis but this aspect has not been really explored yet. Interestingly, lysozyme activity was recently uncovered in the *Ruminococcus champellensis* cellulosome (Morais et al., 2016) and several dockerin-containing CWHs enable to associate to the cohesion domains of a scaffoldin were further reported (Bensoussan et al., 2017). Beyond the cellulosome dedicated to the degradation of plant materials (Shoham et al., 1999; Desvaux, 2005; Smith et al., 2017), a new concept could emerge, that is an extracellular multi-enzymes complex dedicated to bacterial cell lysis, i.e. the lyticome. Clearly, our knowledge of the full diversity of CWHs is still incomplete and new conserved domains will most likely be uncovered in the years to come. Yet more complex are the regulation and modulation of expression as well as activity of CWHs. These secreted enzymes are not only regulated at the transcriptional level but also at different translational and post-translational levels, including translocational and conformational levels, which are the first regulatory levels. A complete understanding of their interactions with CW components and of enzymatic interplay is most certainly the next frontier for breakthroughs in the field, without counting on the many side effects of these enzymes on the bacterial ecophysiology, a topic of much current interest (Wyckoff et al., 2012).

## Author Contributions

AV and MD wrote the first overall draft of the manuscript and drew the original pictures. SL, RT, MP, and CP wrote sections of the manuscript. MD, SL, and RT contributed to conceptualize the overarching aims. MD had management as well as coordination responsibility for the execution of the work. MD and CP contributed to the acquisition of the financial supports and resources leading to this publication. All authors contributed to the critical revision of the manuscript, read and approved the submitted version.

### Conflict of Interest Statement

CP is permanent employee of BioFilm Control and declare his company provided support in the form of salary. The remaining authors declare that the research was conducted in the absence of any commercial or financial relationships that could be construed as a potential conflict of interest.
